# Dose-Response Associations of Lipid Traits With Coronary Artery Disease and Mortality

**DOI:** 10.1001/jamanetworkopen.2023.52572

**Published:** 2024-01-19

**Authors:** Guoyi Yang, Amy M. Mason, Angela M. Wood, C. Mary Schooling, Stephen Burgess

**Affiliations:** 1 School of Public Health, Li Ka Shing Faculty of Medicine, The University of Hong Kong, Hong Kong, China; 2 Medical Research Council Biostatistics Unit, University of Cambridge, Cambridge, United Kingdom; 3 British Heart Foundation Cardiovascular Epidemiology Unit, Department of Public Health and Primary Care, University of Cambridge, Cambridge, United Kingdom; 4 Victor Phillip Dahdaleh Heart and Lung Research Institute, University of Cambridge, Cambridge, United Kingdom; 5 Graduate School of Public Health and Health Policy, City University of New York, New York

## Abstract

**Importance:**

Apolipoprotein B (apoB), low-density lipoprotein cholesterol (LDL-C), and triglycerides (TG) are associated with coronary artery disease (CAD). However, trial evidence for the association of intensive LDL-C lowering and TG lowering with mortality is less definitive.

**Objectives:**

To investigate the associations of apoB, LDL-C, and TG with CAD and mortality, both overall and by sex and age, and to characterize the shapes of these associations.

**Design, Setting, and Participants:**

This genetic association study used linear and nonlinear mendelian randomization (MR) to analyze a population-based cohort of individuals of European ancestry from the UK Biobank, which recruited participants from 2006 to 2010 with follow-up information updated until September 2021. Data analysis occurred from December 2022 to November 2023.

**Exposures:**

Genetically predicted apoB, LDL-C, and TG.

**Main Outcomes and Measures:**

The primary outcomes were CAD, all-cause mortality, and cause-specific mortality. Genetic associations with CAD were calculated using logistic regression, associations with all-cause mortality using Cox proportional hazards regression, and associations with cause-specific mortality using cause-specific Cox proportional hazards regression with censoring for other causes of mortality.

**Results:**

This study included 347 797 participants (mean [SD] age, 57.2 [8.0] years; 188 330 female [54.1%]). There were 23 818 people who developed CAD and 23 848 people who died. Genetically predicted apoB was positively associated with risk of CAD (odds ratio [OR], 1.65 per SD increase; 95% CI 1.57-1.73), all-cause mortality (hazard ratio [HR], 1.11; 95% CI, 1.06-1.16), and cardiovascular mortality (HR, 1.36; 95% CI, 1.24-1.50), with some evidence for larger associations in male participants than female participants. Findings were similar for LDL-C. Genetically predicted TG was positively associated with CAD (OR, 1.60; 95% CI 1.52-1.69), all-cause mortality (HR, 1.08; 95% CI, 1.03-1.13), and cardiovascular mortality (HR, 1.21; 95% CI, 1.09-1.34); however, sensitivity analyses suggested evidence of pleiotropy. The association of genetically predicted TG with CAD persisted but it was no longer associated with mortality outcomes after controlling for apoB. Nonlinear MR suggested that all these associations were monotonically increasing across the whole observed distribution of each lipid trait, with no diminution at low lipid levels. Such patterns were observed irrespective of sex or age.

**Conclusions and relevance:**

In this genetic association study, apoB (or, equivalently, LDL-C) was associated with increased CAD risk, all-cause mortality, and cardiovascular mortality, all in a dose-dependent way. TG may increase CAD risk independent of apoB, although the possible presence of pleiotropy is a limitation. These insights highlight the importance of apoB (or, equivalently, LDL-C) lowering for reducing cardiovascular morbidity and mortality across its whole distribution.

## Key Points

**Question:**

Do apolipoprotein B (apoB), low-density lipoprotein cholesterol (LDL-C), and triglycerides (TG) increase risk of coronary artery disease (CAD), all-cause mortality, or cause-specific mortality, and if so, what are the shapes of these associations?

**Findings:**

In this genetic association study using mendelian randomization including 347 797 participants of European ancestry from UK Biobank, genetically predicted apoB and LDL-C were positively associated with CAD, all-cause mortality, and cardiovascular mortality, all in a dose-dependent way. Genetically predicted TG was positively associated with CAD, although the presence of pleiotropy was suggested.

**Meaning:**

These findings suggest that lowering apoB (or, equivalently, LDL-C) may be associated with reduced cardiovascular morbidity and mortality across its whole observed distribution.

## Teaser

This genetic association study using mendelian randomization examines the association of various lipids (apolipoprotein B, low-density lipoprotein cholesterol, and triglycerides) with coronary artery disease and mortality outcomes.

## Introduction

Lipid management is essential for preventing cardiovascular morbidity and mortality.^1,2^ Apolipoprotein B (apoB) is emerging as the predominant trait that accounts for the etiological associations of lipid traits with coronary artery disease (CAD).^3^ An association of low-density lipoprotein cholesterol (LDL-C) with CAD is widely accepted,^4^ and an association of triglycerides (TG) with CAD is also gaining acceptance.^5,6^ However, dose-response associations of these lipid traits with CAD and mortality remain unclear.

Randomized clinical trials (RCTs) have shown that lowering LDL-C reduces cardiovascular disease (CVD) events and mortality,^7^ and intensive LDL-C lowering further reduces CVD events compared with standard LDL-C lowering.^8,9,10^ However, no reduction in all-cause mortality or CVD mortality has been shown in trials comparing more intensive and less intensive statin therapy,^11,12,13^ or in trials adding PCSK9 (proprotein convertase subtilisin and kexin type 9) inhibitors^9^ or ezetimibe^10^ to background statin therapy. Meta-regression of RCTs suggests the magnitude of LDL-C lowering is associated with greater reduction in all-cause mortality and CVD mortality in trials of people with higher baseline LDL-C.^14^ RCTs have shown therapies primarily lowering TG (ie, fibrates and omega-3 supplements) reduce CVD events but have little detectable effect on all-cause mortality or CVD mortality.^15,16^ Previous studies have shown adverse associations of lipid modifiers with myopathy, liver enzyme elevation, and type 2 diabetes.^17,18,19^ Taken together, these studies raise the question about the balance of risks and benefits for lipid lowering, particularly for groups with low baseline LDL-C or TG, as well as for women (who have lower CVD risk than men), and for older people (who generally have more non-CVD comorbidities than younger people).

Observational studies have shown J-shaped associations of LDL-C and TG with all-cause mortality.^20,21^ However, the shape of these associations could also be an indicator of confounding or selection bias. Better understanding of the shape of associations of lipid traits with CAD and mortality has clinical implications for determining lipid-lowering goals for CVD prevention.

Mendelian randomization (MR), an instrumental variable analysis with genetic instruments, takes advantage of genetic randomization at conception to obtain less confounded estimates than conventional observational studies.^22^ MR relies on the instrumental variable assumptions of relevance, independence, and exclusion restriction (ie, genetic instruments should be associated with the exposure, share no common cause with the outcome, and be independent of the outcome given the exposure).^22^ Previous MR studies^3,5,23^ have suggested apoB, LDL-C, and TG are positively associated with risk of CAD and all-cause mortality.^24^ However, these studies^3,5,23,24^ did not take into account potential nonlinearity and differences by sex or age. To address the gap, we first conducted linear MR analyses to assess the associations of genetically predicted apoB, LDL-C, and TG with CAD, all-cause mortality, and cause-specific mortality. We then performed nonlinear MR analyses to characterize the shape of these associations. We conducted subgroup analyses by sex because sex-specific outcomes are evident for LDL-C,^25^ TG,^26^ and some lipid modifiers.^27^ We also conducted subgroup analyses by age (<65 years and ≥65 years) because of potential concerns about efficacy of lipid lowering in older people.^28^

## Methods

### Ethical Approval of Studies and Informed Consent

This genetic association study did not require institutional review board approval because it was an analysis of publicly available summary statistics and was not considered human participants research in accordance with the Common Rule. This study followed the Strengthening the Reporting of Observational Studies in Epidemiology STROBE MR reporting guideline,^29^ and was conducted using the UK Biobank Resource. The UK Biobank obtained ethical approval from the North West Multicenter Research Ethics Committee, and the participants provided written informed consent.

### Study Design

We performed linear and nonlinear MR analyses in UK Biobank. The UK Biobank recruited approximately 500 000 people (intended age, 40-69 years; 94% self-reported European ancestry) from 2006 to 2010 in England, Scotland, and Wales.^30^ Participants completed a range of physical assessments and questionnaires including socioeconomic characteristics, lifestyle, and health-related conditions; they also provided samples for biological measurement and genotyping.^30^ Follow-up information was obtained by linking to national medical and mortality records.^30^ We performed analyses using data updated until September 2021. We included unrelated individuals of European ancestry with valid measurements of apoB, LDL-C, and TG at baseline, and genomic data passing quality control as described previously.^31^

CAD was defined on the basis of both prevalent cases (self-reported health conditions and medical records at recruitment) and incident events (*International Statistical Classification of Diseases and Related Health Problems, Tenth Revision [ICD-10]* codes I20-I25) during the follow-up. All-cause mortality was determined by the participant’s mortality status, and cause-specific mortality was divided into CVD mortality, cancer mortality, and non-CVD or cancer mortality using *ICD-10* codes (eTable 1 in Supplement 1). We replicated the findings for all-cause mortality using parental mortality status from European descent summary statistics of a meta-analysis^32^ of UK Biobank and LifeGen (1 012 240 individuals; 609 139 [60%] deceased) to reduce selection bias and increase power.

### Statistical Analysis

All statistical analyses were conducted using R statistical software version 4.2.1 (R Project for Statistical Computing) with the packages ieugwasr, MendelianRandomization, and SUMnlmr. Data analysis occurred from December 2022 to November 2023.

#### Genetic Risk Score for ApoB, LDL-C, and TG

We extracted 163 independent (*r*^2^ < 0.001) genome-wide significant (*P* < 5 × 10^−8^) genetic instruments for apoB from a genome-wide association study (GWAS) of individuals of White British genetic ancestry from the UK Biobank.^33^ We also extracted 313 genetic instruments for LDL-C and 373 genetic instruments for TG from European-descent summary statistics in Global Lipids Genetics Consortium,^34^ excluding the UK Biobank participants.

We generated genetic risk scores (GRSs) for apoB, LDL-C, and TG. For each lipid trait, we multiplied the number of lipid-increasing alleles by variant-specific association from the original GWAS and summed across all the genetic variants. We calculated the proportion of variance explained by the GRS and the *F* statistic to assess instrument strength. We also checked whether the GRSs were associated with possible confounders (ie, Townsend Deprivation Index, current smoking, current alcohol drinking, and physical activity).

#### Linear MR

We calculated linear MR estimates using the ratio method by dividing the association of the GRS with the outcome by the association of the GRS with the exposure. We transformed each lipid trait using inverse rank-normalization for comparability, and obtained genetic associations with apoB, LDL-C, and TG using linear regression. We obtained genetic associations with CAD using logistic regression, associations with all-cause mortality using Cox proportional hazards regression, and associations with cause-specific mortality using cause-specific Cox proportional hazards regression with censoring for other causes of mortality. We adjusted for baseline age, baseline age squared, sex, baseline age by sex, baseline age squared by sex, and the first 20 principal components for genetic associations with lipid traits and CAD. We used attained age as the time variable in Cox regression models, and adjusted for birth year, birth year squared, sex, birth year by sex, birth year squared by sex, and the first 20 principal components for genetic associations with mortality outcomes. To assess the robustness of ratio estimates, we conducted sensitivity analyses using methods with different assumptions about instrumental validity (ie, inverse-variance weighted [IVW],^35^ weighted median,^36^ MR Egger,^37^ and contamination mixture methods).^38^

We used multivariable MR to assess the association of each lipid trait controlling for potential pleiotropy.^39^ For each multivariable MR model, we combined all the genetic instruments, dropped duplicated single nucleotide variants (SNVs), and removed correlated (*r^2^* ≥ 0.001) SNVs. We used the remaining SNVs to generate GRSs for apoB, LDL-C, and TG, taking weights from the original GWAS. Given the high correlation between apoB and LDL-C (*r^2^* = 0.96), we adjusted for genetically predicted TG in the calculation of apoB and LDL-C estimates. Because apoB is emerging as the predominant trait in the cause of CAD,^3,40^ we adjusted for genetically predicted apoB in the calculation of TG estimates. We calculated the conditional *F* statistic to assess instrument strength to estimate each trait conditioning on the other trait in multivariable MR.^41^ We used multivariable IVW and multivariable MR Egger as sensitivity analyses. As further sensitivity analyses, we additionally included possible confounders associated with the GRS (*P* < .01) in multivariable MR analyses.

#### Nonlinear MR

We applied the fractional polynomial method to examine nonlinear associations.^42^ We stratified the population into 10 strata using the doubly ranked method.^43^ We first stratified the population into preliminary strata according to the instrument level, and then stratified them into final strata on the basis of the exposure level within each prestratum.^43^ For each stratum of the population, the instrument remained independent of confounders, and a linear MR estimate was calculated using the ratio method as described above. We did not perform inverse rank-normalization transformation for each lipid trait in nonlinear MR analyses because RCTs suggest clinical benefits of statin therapy are determined by absolute reduction in LDL-C.^7^ We meta-regressed the linear MR estimates against the mean value of the exposure in each stratum.^42^ We used a trend test to assess whether a linear trend in the stratum-specific estimates existed, and a fractional polynomial test to examine whether a nonlinear model fit the exposure-outcome association better than a linear model.^42^ Differences by sex were assessed using a 2-sided *z* test.^44^ Statistical significance was defined as 2-sided *P* < .05.

## Results

### Baseline Characteristics

This study included 347 797 participants (mean [SD] age, 57.2 [8.0] years; 188 330 female [54.1%]) (Table). There were 23 818 people who developed CAD (including 17 136 prevalent cases), and 23 848 people who died. The GRSs explained 12.1% of the variance in apoB (*F* = 1835), 10.7% of the variance in LDL-C (*F* = 1610), and 14.8% of the variance in TG (*F* = 2315). An *F* statistic greater than 10 indicated that bias from weak instruments was minimal. The GRSs for apoB (odds ratio [OR], 0.95; 95% CI, 0.91-0.98; *P* = .005) and LDL-C (OR, 0.93; 95% CI, 0.89-0.97; *P* = .002) were inversely associated with current smoking. The GRS for TG was inversely associated with current alcohol drinking (OR, 0.92; 95% CI, 0.87-0.96; *P* = .001) (eTable 2 in Supplement 1).

**Table.** Baseline Characteristics of Participants in the UK Biobank

Characteristic	Participants, No. (%) (N = 347 797)
Age at recruitment, y	
Mean (SD)	57.2 (8.0)
<65	281 076 (80.8)
≥65	66 721 (19.2)
Sex	
Male	159 467 (45.9)
Female	188 330 (54.1)
Apolipoprotein B, mean (SD), mg/dL	103.4 (23.8)
Low-density lipoprotein cholesterol, mean (SD), mg/dL	137.8 (33.2)
Triglycerides, median (IQR) mg/dL	131.2 (92.7-189.4)
Body mass index, mean (SD)^a^	27.4 (4.8)
Systolic blood pressure, mean (SD), mm Hg	137.6 (18.6)
Townsend Deprivation Index score, mean (SD)	−1.5 (3.0)
Metabolic equivalent task score, median (IQR), min/wk	1782 (813-3568)
Smoking	
Current	35 727 (10.3)
Other	312 070 (89.7)
Alcohol drinking	
Current	324 289 (93.2)
Other	23 508 (6.8)
Lipid-lowering medication	
Current	58 851 (16.9)
Other	288 946 (83.1)
Coronary artery disease	23 818 (6.8)
Deaths	23 848 (6.9)
Cardiovascular disease deaths	4944 (1.4)
Cancer deaths	12 247 (3.5)
Noncardiovascular disease or cancer deaths	6427 (1.8)

To convert apolipoprotein B to grams per liter, multiply by .01; low-density lipoprotein cholesterol to millimoles per liter, multiply by 0.0259; and triglycerides to millimoles per liter, multiply by .0113.

^a^ Body mass index was calculated as weight in kilograms divided by height in meters squared.

### Linear MR Analyses

**Figure 1.**
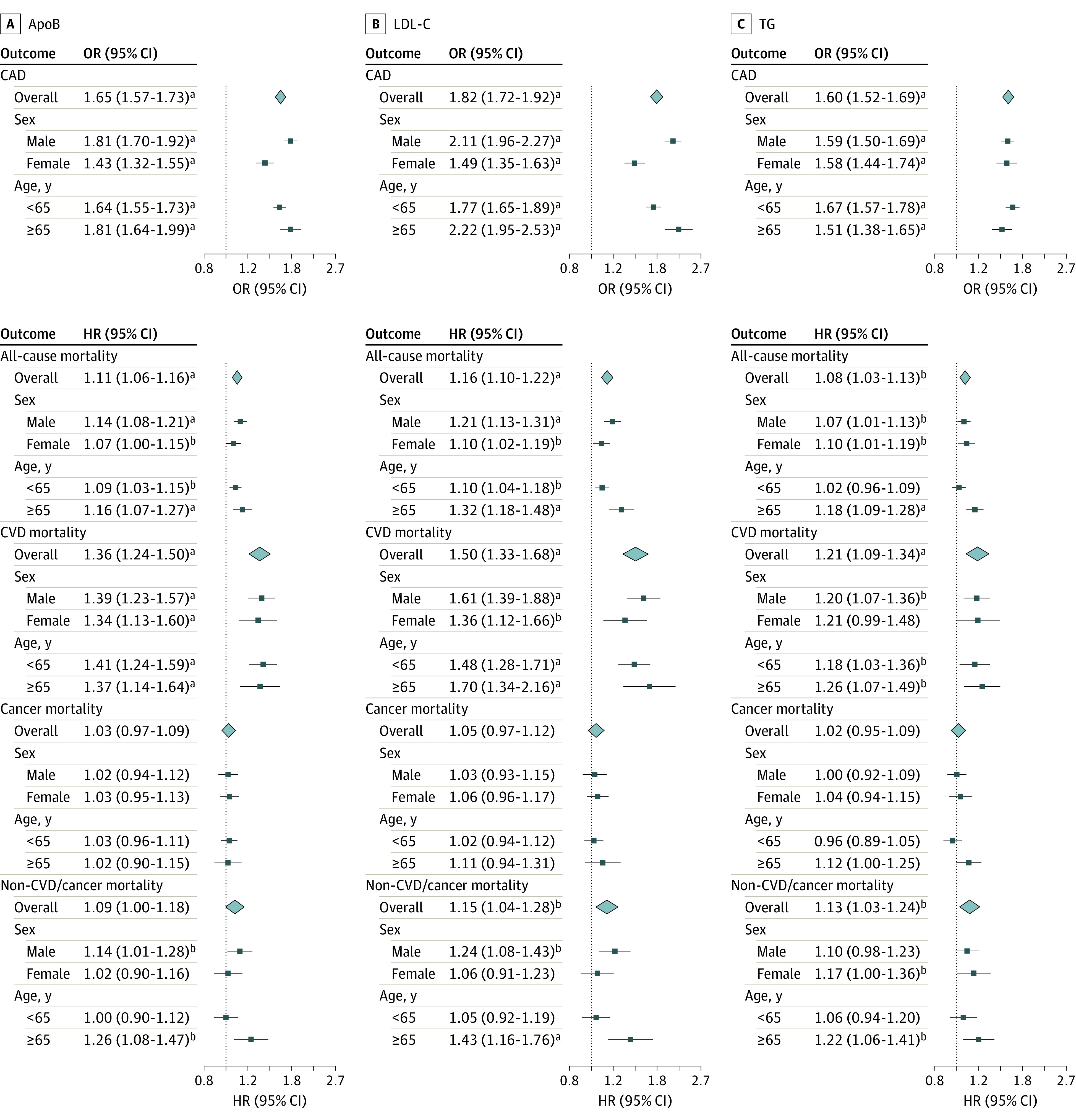
Univariable Estimates for Genetically Predicted Lipid Traits and Risk of Coronary Artery Disease (CAD), All-Cause Mortality, and Cause-Specific Mortality The figure shows univariable estimates for apolipoprotein B (apoB)*,* low-density lipoprotein cholesterol (LDL-C), and triglycerides (TG). Estimates are expressed as odds ratios (ORs) and hazard ratios (HRs) with 95% CIs per SD increase in genetically predicted level of each lipid trait (approximately 23.8 mg/dL for apoB [to convert to grams per liter, multiply by .01], 33.2 mg/dL [to convert to millimoles per liter, multiply by 0.0259] for LDL-C, and 87.5 mg/dL [to convert to millimoles per liter, multiply by .0113] for TG). CVD indicates cardiovascular disease. Squares denote stratum estimates, and diamonds denote overall estimates. Error bars denote 95% CIs. ^a^*P* < .001. ^b^*P* < .05.

In univariable MR, genetically predicted apoB was positively associated with risk of CAD (OR per SD increase, 1.65; 95% CI, 1.57-1.73), all-cause mortality (hazard ratio [HR], 1.11; 95% CI, 1.06-1.16), and CVD mortality (HR, 1.36; 95% CI, 1.24-1.50), with some evidence for larger associations in male participants than female participants (CAD OR for male participants, 1.81; 95% CI, 1.70-1.92 vs OR for female participants, 1.43; 95% CI, 1.32-1.55; *P *for difference < .001; all-cause mortality HR for male participants, 1.14; 95% CI, 1.08-1.21 vs HR for female participants, 1.07; 95% CI, 1.00-1.15; *P* for difference = .17; CVD mortality HR for male participants, 1.39; 95% CI, 1.23-1.57 vs HR for female participants, 1.34; 95% CI, 1.13-1.60; *P* for difference = .76) (Figure 1). Genetically predicted apoB was not associated with cancer mortality but had a positive association with non-CVD or cancer mortality in male participants and older people (Figure 1). Similar patterns were observed for LDL-C (Figure 1). Genetically predicted TG was positively associated with risk of CAD (OR, 1.60; 95% CI, 1.52-1.69), all-cause mortality (HR, 1.08; 95% CI, 1.03-1.13), CVD mortality (HR, 1.21; 95% CI, 1.09-1.34), and non-CVD or cancer mortality (HR, 1.13; 95% CI, 1.03-1.24) (Figure 1). Sensitivity analyses using other analytic methods gave similar estimates for apoB and LDL-C; however, the MR Egger intercept indicated directional pleiotropy for TG on CAD, and estimates for TG were attenuated in weighted median and MR Egger methods (eTable 3 in Supplement 1).

**Figure 2.**
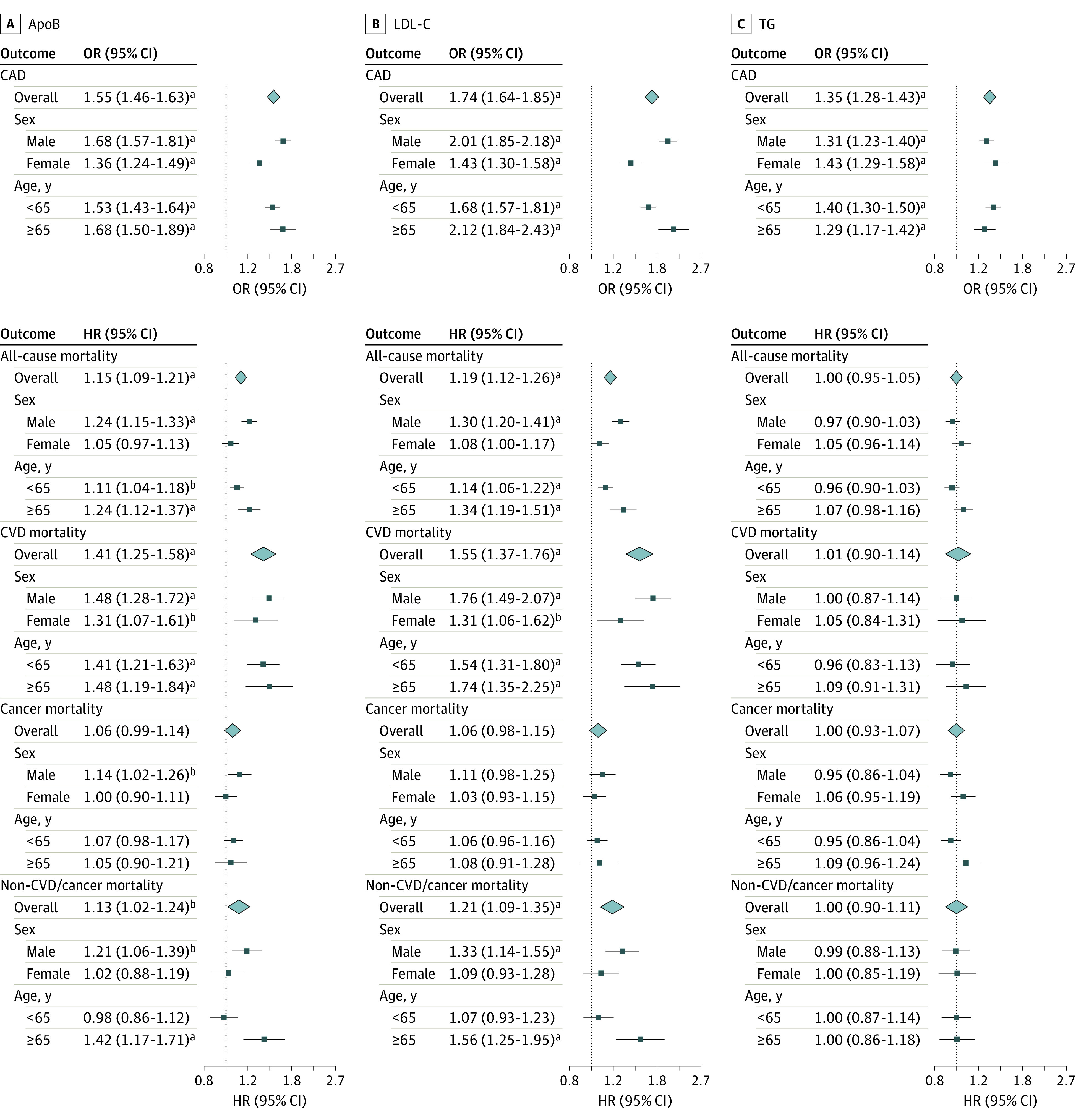
Multivariable Estimates for Genetically Predicted Lipid Traits and Coronary Artery Disease (CAD), All-Cause Mortality, and Cause-Specific Mortality The figure shows multivariable estimates for apolipoprotein B (apoB*),* low-density lipoprotein cholesterol (LDL-C), and triglycerides (TG). Multivariable estimates for apoB and LDL-C were adjusted for TG, and multivariable estimates for TG were adjusted for apoB. Estimates are expressed as odds ratios (ORs) and hazard ratios (HRs) with 95% CIs per standard deviation increase in genetically predicted level of each lipid trait (approximately 23.8 mg/dL for apoB [to convert to grams per liter, multiply by .01], 33.2 mg/dL [to convert to millimoles per liter, multiply by 0.0259] for LDL-C, and 87.5 mg/dL [to convert to millimoles per liter, multiply by .0113] for TG). CVD indicates cardiovascular disease. Squares denote stratum estimates, and diamonds denote overall estimates. Error bars denote 95% CIs. ^a^*P* < 001. ^b^*P* < .05.

In multivariable MR, the conditional *F* statistic^45^ indicated that bias from weak instruments was minimal for apoB (*F* = 70), LDL-C (*F* = 51), and TG (*F* = 73). After controlling for TG, the associations of genetically predicted apoB and LDL-C were similar to those in univariable MR (Figure 2). Although multivariable MR controlling for apoB showed some attenuation in the association of genetically predicted TG with CAD, the positive association persisted (Figure 2). However, after controlling for apoB*,* there was no longer an association of genetically predicted TG with all-cause mortality, CVD mortality, or non-CVD or cancer mortality (Figure 2). Similar results were obtained when using multivariable IVW and multivariable MR Egger, as well as additionally including possible confounders (ie, current smoking for apoB and LDL-C and current alcohol drinking for TG) (eTable 4 in Supplement 1).

For replication using parental mortality status, genetically predicted apoB, LDL-C, and TG were positively associated with parental all-cause mortality in univariable MR, although the MR Egger intercept indicated directional pleiotropy for TG (eTable 5 in Supplement 1). Multivariable MR controlling for TG gave similar results for apoB and LDL-C; however, multivariable MR controlling for apoB attenuated the association of TG with parental all-cause mortality (eTable 6 in Supplement 1).

### Nonlinear MR Analyses

**Figure 3.**
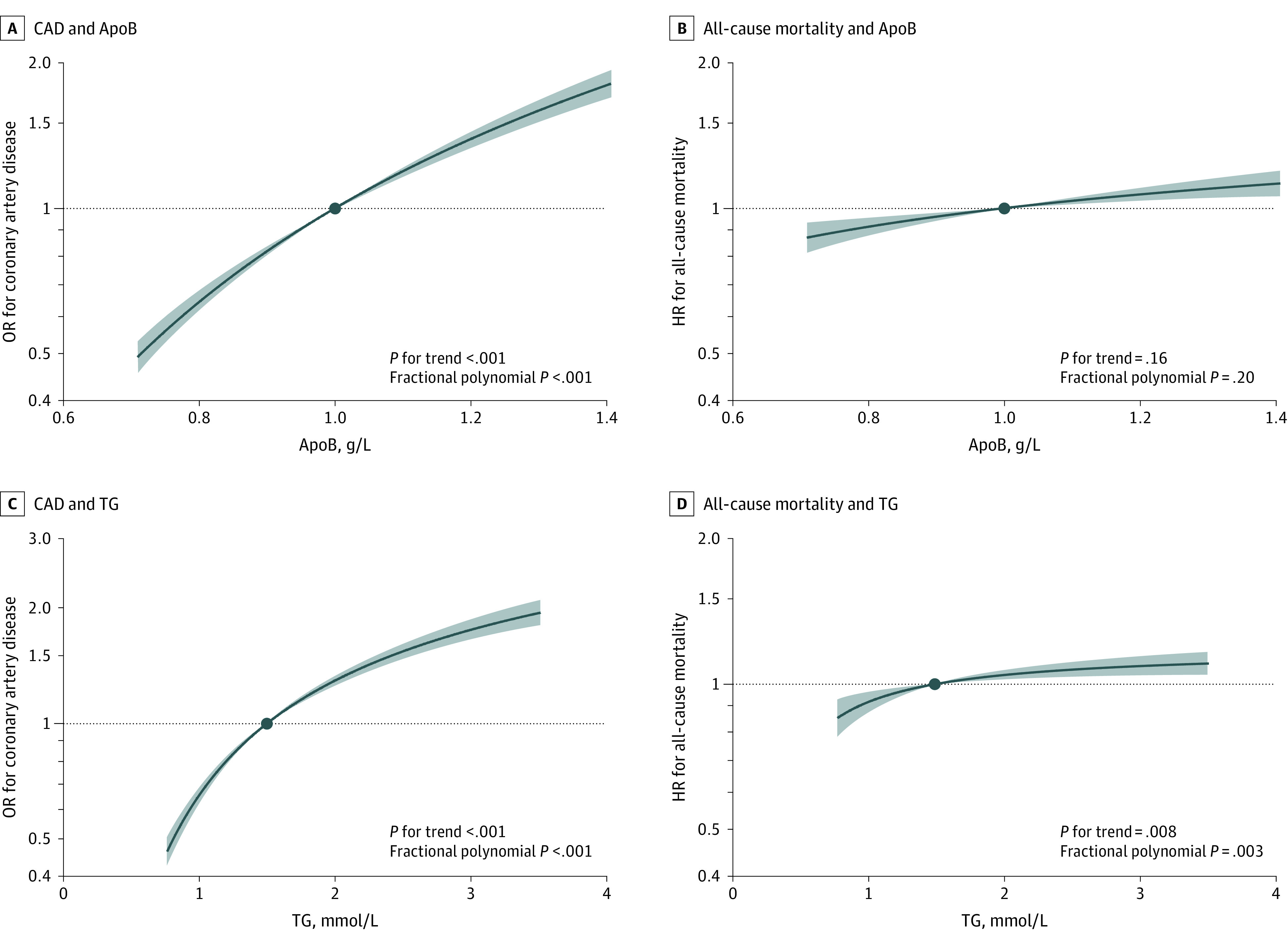
Shapes of the Associations of Genetically Predicted Apolipoprotein B (apoB) and Triglycerides (TG) With Coronary Artery Disease (CAD) and All-Cause Mortality The x-axis depicts apoB level in g/L or TG level in mmol/L. The y-axis depicts the odds ratio (OR) for CAD or hazard ratio (HR) for all-cause mortality with respect to the reference, plotted on a log scale. Reference is set to the median of each lipid trait (1.0 g/L for apoB and 1.5 mmol/L for TG). The black lines represent the dose-response association, and the blue shading represent the 95% CIs. The trend test assesses whether a linear trend in the stratum-specific estimates exists, and the fractional polynomial test examines whether a nonlinear model fits the exposure-outcome association better than a linear model.

We observed monotonically increasing associations of genetically predicted apoB with CAD, all-cause mortality, and CVD mortality (Figure 3 and eFigure 1 in Supplement 1). Although nonlinear MR suggested a smaller association of genetically predicted apoB with CAD risk as apoB increased, stratum-specific associations remained positive across the whole distribution of apoB (eTable 7 in Supplement 1). There was no statistical evidence favoring a nonlinear association of genetically predicted apoB with mortality outcomes over a linear one (eFigure 1 in Supplement 1). Similar patterns were observed for LDL-C (eFigure 1 and eTable 8 in Supplement 1). Genetically predicted TG had monotonically increasing associations with CAD, all-cause mortality, CVD mortality, and non-CVD or cancer mortality (Figure 3 and eFigure 1 in Supplement 1). These associations were lessened with increasing TG, but stratum-specific associations were generally positive across the distribution of TG (eTable 9 in Supplement 1). Nonlinear MR analyses by sex and age showed similar shapes of associations as in the whole population (eFigures 2-5 in Supplement 1).

## Discussion

Consistent with RCTs^7,8,15,16^ and previous MR studies,^3,5,23,24^ in this genetic association study, we found positive associations of genetically predicted apoB and LDL-C with CAD, all-cause mortality, and CVD mortality, and genetically predicted TG was positively associated with CAD. Our investigation has added to the evidence base by showing such associations hold across the whole distribution of apoB, LDL-C, or TG.

Our findings provide genetic evidence suggesting no threshold of lowering apoB or, equivalently, LDL-C (ie, the main apoB-containing lipoprotein) for reducing risk of CAD, all-cause mortality, and CVD mortality, further supporting the concept of the lower the better. The retention of apoB-containing lipoproteins in the artery wall and subsequent release of cholesterol contents are essential for the initiation and progression of atherosclerosis.^46^ Our findings are consistent with RCTs^8,9,10^ showing that intensive LDL-C lowering further reduces CVD events compared with standard LDL-C lowering. However, most RCTs^9,10,11,12,13^ failed to show significant incremental benefits of intensive LDL-C lowering on all-cause mortality or CVD mortality. This discrepancy may be related to insufficient difference in LDL-C between groups, short follow-up duration, and low proportion of death from CVD in RCTs,^9,10,11,12,13^ or differing effects on mortality between LDL-C lowering therapies.^47^ Notably, a trial^48^ has shown high-dose statin therapy further reduces all-cause mortality compared with low-dose therapy in Japanese patients with the mean LDL-C less than 90 mg/dL (to convert to mmol/L, multiply by 0.0259) at baseline. Our findings are consistent with a recent MR study^49^ showing no threshold in the association of LDL-C with CAD, although that study used a different approach for instrument selection and lacked power to detect a significant association of LDL-C with all-cause mortality.

The decreasing association of genetically predicted LDL-C with CAD risk as LDL-C increases should be interpreted cautiously because fewer individuals with higher LDL-C survived to be recruited^50^ and more of them died during follow-up,^51^ which may lead to an artifact of less harmful effects. Prevalent statin use could mitigate the genetic association of LDL-C with CAD risk, particularly in people with higher LDL-C.^52^ It is also possible that the decreasing association reflects effect heterogeneity in population groups with different demographic characteristics rather than nonlinearity for any specific individual. Furthermore, LDL-C confers greater absolute risk of CAD in people with higher LDL-C than people with lower LDL-C, even for the same relative risk.

Despite consensus about the benefits of LDL-C lowering, previous MR studies^53,54^ showed LDL-C lowering may increase risk of type 2 diabetes^19^ and some cancers. However, we found no adverse association of LDL-C lowering with cancer mortality or other (ie, non-CVD or cancer) mortality, although we cannot exclude the possibility that LDL-C lowering may increase specific cancer risk. Consistently, a 2023 study^55^ showed long-term achievement of lower LDL-C levels (down to 20 mg/dL) is associated with lower CVD risk without significant safety concerns. Taken together, these findings suggest the cardiovascular benefits of LDL-C lowering outweigh its adverse effects, which results in a reduction in all-cause mortality, even in people with low baseline LDL-C.

Male participants demonstrated a larger association of genetically predicted LDL-C with CAD (*P* for difference < .001) than female participants, which implies that male individuals might benefit more from LDL-C lowering therapies than female individuals. Our findings are consistent with a 2022 MR study^25^ showing sex-specific associations of LDL-C with CVD. However, a meta-analysis^7^ of RCTs suggested similar beneficial effects of LDL-C lowering by statins on major CVD events and all-cause mortality for women and men, possibly attributed to underrepresentation of women (27%) in statin trials. Further investigations are warranted to understand potential factors underlying sex-specific effects of LDL-C, such as sex hormones.^56^

Our findings for TG are intriguing. In univariable MR, genetically predicted TG was positively associated with risk of CAD, all-cause mortality, and CVD mortality. However, we found evidence of directional pleiotropy, which suggests potential bias or reflects different biological mechanisms involved in any effects of TG. In multivariable MR, which approximates the scenario for changing TG without altering apoB, the association of TG with CAD remained positive, but there were no longer associations with all-cause mortality and CVD mortality. Correspondingly, RCTs^15,16^ show therapies primarily lowering TG (ie, fibrates and omega-3 supplements) reduce CVD events but have little effect on mortality. It is also possible that TG lowering may affect CVD diagnosis, and thus its beneficial effect on CVD is an artifact mainly associated with reduction in nonfatal events, such as revascularization.^57^

### Limitations

This is, to our knowledge, the first MR study comprehensively assessing associations of lipid traits with CAD, all-cause mortality, and cause-specific mortality, taking into account potential nonlinearity as well as differences by sex and age. Nevertheless, this study has several limitations. First, MR relies on 3 rigorous assumptions of relevance, independence, and exclusion restriction.^22^ The GRSs were inversely associated with current smoking or alcohol drinking, possibly due to pleiotropy or selecting on survival before recruitment. We addressed this potential bias by using multivariable MR.^39,58^ Second, we used multivariable MR to assess the association of apoB (or equivalently LDL-C) adjusted for TG and vice versa, as they might have bidirectional associations. Adjustment for apoB might have inappropriately masked some effects of TG, because the clinical benefits of lowering TG could be associated with reduction in apoB^40^ or with other mechanisms.^26^ Further investigation is needed to elucidate the underlying mechanisms of the association of TG with CAD and mortality. Third, we conducted nonlinear MR analyses to characterize the shapes of associations. However, due to a current lack of suitable methods, we were unable to perform nonlinear multivariable MR analyses. Fourth, we obtained genetic instruments for apoB from the same study as genetic associations with CAD and mortality. Bias due to participant overlap depends on the strength of genetic associations with the exposure,^59^ and thus should not be substantial given the high *F* statistic for apoB. Fifth, we calculated the GRSs for apoB, LDL-C, and TG from GWAS performed in a whole population and applied them to derive sex-specific and age-specific genetic associations. However, it is unlikely to have changed our results substantially given the genetics of most biomarkers are shared between women and men.^60^ Any minor discrepancies between overall estimates and the weighted average of subgroup estimates are likely due to noncollapsibility of ORs or HRs. Sixth, the analyses were restricted to people of European ancestry and may not apply to other populations. Although we found monotonically increasing associations of LDL-C with CAD, all-cause mortality, and CVD mortality across the observed distribution of LDL-C, we cannot exclude the possibility of a threshold association in populations with relatively low LDL-C, such as East Asian populations.^61^ A previous MR study showed LDL-C is inversely associated with intracerebral hemorrhage in East Asian individuals,^62^ which may detract from benefits of LDL-C lowering or could be due to selection bias. Seventh, MR assesses lifelong effects of lipid traits, which cannot directly inform the quantitative effects of lipid-lowering therapies in the short term.

## Conclusions

This genetic association study suggests that apoB (or, equivalently, LDL-C) is associated with increased risk of CAD, all-cause mortality, and CVD mortality in a dose-dependent way. TG may be associated with increased CAD risk independently of apoB, but the possible presence of pleiotropy is a limitation. These insights highlight the importance of apoB (or, equivalently, LDL-C) lowering for reducing CVD morbidity and mortality across its whole distribution.

## Notes

Supplement 1. **eTable 1.** *International Statistical Classification of Diseases and Related Health Problems, Tenth Revision (ICD-10)* Codes for Cause-Specific Mortality Outcomes **eTable 2.** Mendelian Randomization Estimates Using the Ratio Method for Genetically-Predicted ApoB, LDL-C, and TG on Possible Confounders **eTable 3.** Univariable Mendelian Randomization Estimates for Genetically-Predicted ApoB, LDL-C, and TG on CAD, All-Cause Mortality, and Cause-Specific Mortality **eTable 4.** Multivariable Mendelian Randomization Estimates for Genetically-Predicted ApoB, LDL-C, and TG on CAD, All-Cause Mortality, and Cause-Specific Mortality Controlling for Other Lipid Traits and Possible Confounders **eTable 5.** Univariable Mendelian Randomization Estimates for Genetically-Predicted ApoB, LDL-C, and TG on Parental All-Cause Mortality **eTable 6.** Multivariable Mendelian Randomization Estimates for Genetically-Predicted ApoB, LDL-C, and TG on Parental All-Cause Mortality Controlling for Other Lipid Traits and Possible Confounders **eFigure 1**. Shapes of the Associations of Genetically-Predicted ApoB, LDL-C, and TG With CAD, All-Cause Mortality, and Cause-Specific Mortality Overall **eTable 7.** Stratum-Specific Mendelian Randomization Estimates for Genetically-Predicted ApoB on CAD, All-Cause Mortality, and Cause-Specific Mortality **eTable 8.** Stratum-Specific Mendelian Randomization Estimates for Genetically-Predicted LDL-C on CAD, All-Cause Mortality, and Cause-Specific Mortality **eTable 9.** Stratum-Specific Mendelian Randomization Estimates for Genetically-Predicted TG on CAD, All-Cause Mortality, and Cause-Specific Mortality **eFigure 2.** Shapes of the Associations of Genetically-Predicted ApoB, LDL-C, and TG With CAD, All-Cause Mortality, and Cause-Specific Mortality for Males **eFigure 3.** Shapes of the Associations of Genetically-Predicted ApoB, LDL-C, and TG With CAD, All-Cause Mortality, and Cause-Specific Mortality for Females **eFigure 4.** Shapes of the Associations of Genetically-Predicted ApoB, LDL-C, and TG With CAD, All-Cause Mortality, and Cause-Specific Mortality for Younger People **eFigure 5.** Shapes of the Associations of Genetically-Predicted ApoB, LDL-C, and TG With CAD, All-Cause Mortality, and Cause-Specific Mortality for Older People

Supplement 2.
Data Sharing Statement
