# Supplementary material for: Dose-Response Associations of Lipid Traits With Coronary Artery Disease and Mortality
Source: JAMA Netw Open. 2024 Jan 19;7(1):e2352572. doi: 10.1001/jamanetworkopen.2023.52572 (PMC10799266; doi:10.1001/jamanetworkopen.2023.52572)
Supplement: Supplement 1. — eTable 1. International Statistical Classification of Diseases and Related Health Problems, Tenth Revision (ICD-10) Codes for Cause-Specific Mortality Outcomes eTable 2. Mendelian Randomization Estimates Using the Ratio Method for Genetically-Predicted ApoB, LDL-C, and TG on Possible Confounders eTable 3. Univariable Mendelian Randomization Estimates for Genetically-Predicted ApoB, LDL-C, and TG on CAD, All-Cause Mortality, and Cause-Specific Mortality eTable 4. Multivariable Mendelian Randomization Estimates for Genetically-Predicted ApoB, LDL-C, and TG on CAD, All-Cause Mortality, and Cause-Specific Mortality Controlling for Other Lipid Traits and Possible Confounders eTable 5. Univariable Mendelian Randomization Estimates for Genetically-Predicted ApoB, LDL-C, and TG on Parental All-Cause Mortality eTable 6. Multivariable Mendelian Randomization Estimates for Genetically-Predicted ApoB, LDL-C, and TG on Parental All-Cause Mortality Controlling for Other Lipid Traits and Possible Confounders eFigure 1. Shapes of the Associations of Genetically-Predicted ApoB, LDL-C, and TG With CAD, All-Cause Mortality, and Cause-Specific Mortality Overall eTable 7. Stratum-Specific Mendelian Randomization Estimates for Genetically-Predicted ApoB on CAD, All-Cause Mortality, and Cause-Specific Mortality eTable 8. Stratum-Specific Mendelian Randomization Estimates for Genetically-Predicted LDL-C on CAD, All-Cause Mortality, and Cause-Specific Mortality eTable 9. Stratum-Specific Mendelian Randomization Estimates for Genetically-Predicted TG on CAD, All-Cause Mortality, and Cause-Specific Mortality eFigure 2. Shapes of the Associations of Genetically-Predicted ApoB, LDL-C, and TG With CAD, All-Cause Mortality, and Cause-Specific Mortality for Males eFigure 3. Shapes of the Associations of Genetically-Predicted ApoB, LDL-C, and TG With CAD, All-Cause Mortality, and Cause-Specific Mortality for Females eFigure 4. Shapes of the Associations of Genetically-Predicted ApoB, LDL-C, and TG [file jamanetwopen-e2352572-s001.pdf]

Supplemental Online Content

Yang G, Mason AM, Wood AM, Schooling CM, Burgess S. Dose-responsed associations of lipid tratis with coronary artery disase and mortality: a linear and nonlinear mendelian randomization study. *JAMA Netw* Open. 2024;7(1):e2352572. doi:10.1001/jamanetworkopen.2023.52572

eTable 1. *International Statistical Classification of Diseases and Related Health Problems, Tenth Revision (ICD-10)* Codes for Cause-Specific Mortality Outcomes

eTable 2. Mendelian Randomization Estimates Using the Ratio Method for Genetically-Predicted apoB, LDL-C, and TG on Possible Confounders

eTable 3. Univariable Mendelian Randomization Estimates for Genetically-Predicted ApoB, LDL-C, and TG on CAD, All-Cause Mortality, and Cause-Specific Mortality

eTable 4. Multivariable Mendelian Randomization Estimates for Genetically-Predicted ApoB, LDL-C, and TG on CAD, All-Cause Mortality, and Cause-Specific Mortality Controlling for Other Lipid Traits and Possible Confounders

eTable 5. Univariable Mendelian Randomization Estimates for Genetically-Predicted ApoB, LDL-C, and TG on Parental All-Cause Mortality

eTable 6. Multivariable Mendelian Randomization Estimates for Genetically-Predicted ApoB, LDL-C, and TG on Parental All-Cause Mortality Controlling for Other Lipid Traits and Possible Confounders

eFigure 1. Shapes of the Associations of Genetically-Predicted ApoB, LDL-C, and TG With CAD, All-Cause Mortality, and Cause-Specific Mortality Overall

eTable 7. Stratum-Specific Mendelian Randomization Estimates for Genetically-Predicted ApoB on CAD, All-Cause Mortality, and Cause-Specific Mortality

eTable 8. Stratum-Specific Mendelian Randomization Estimates for Genetically-Predicted LDL-C on CAD, All-Cause Mortality, and Cause-Specific Mortality

eTable 9. Stratum-Specific Mendelian Randomization Estimates for Genetically-Predicted TG on CAD, All-Cause Mortality, and Cause-Specific Mortality

eFigure 2. Shapes of the Associations of Genetically-Predicted ApoB, LDL-C, and TG With CAD, All-Cause Mortality, and Cause-Specific Mortality for Males

eFigure 3. Shapes of the Associations of Genetically-Predicted ApoB, LDL-C, and TG With CAD, All-Cause Mortality, and Cause-Specific Mortality for Females

© 2024 Yang G et al. *JAMA Network Open.*

eFigure 4. Shapes of the Associations of Genetically-Predicted ApoB, LDL-C, and TG With CAD, All-Cause Mortality, and Cause-Specific Mortality for Younger People

eFigure 5. Shapes of the Associations of Genetically-Predicted ApoB, LDL-C, and TG With CAD, All-Cause Mortality, and Cause-Specific Mortality for Older People

This supplemental material has been provided by the authors to give readers additional information about their work.

eTable 1. *International Statistical Classification of Diseases and Related Health Problems, Tenth Revision (ICD-10)* Codes for Cause-Specific Mortality Outcomes

Cause-specific mortality	ICD-10 codes
CVD	G45, I01, I03-I82, I87, I95-I99, F01, Q20-Q28, R96
Cancer	C00-C97, D00-D48
Non-CVD/cancer	A00-A99, B00-B99, D50-D99,E00-E99, F00, F02-F99, G00-G44, G46-G99, H00-H99, I00, I02, I83-I86, I88-I89, J00-J99, K00-K99, L00-L99, M00-M99, N00-N99, O00-O99, P00-P99, Q00-Q18, Q30-Q99, S00-S99, T00-T99, U04, V00-V99, W00-W99, X00-X99, Y00-Y99, Z00-Z99

CVD, cardiovascular disease; ICD, International Classification of Diseases.

eTable 2. Mendelian Randomization Estimates Using the Ratio Method for Genetically-Predicted ApoB, LDL-C, and TG on Possible Confounders

Exposure	Outcome	Measure	Estimates	*P* value
ApoB	Townsend deprivation index	Beta	-0.04 (-0.07 to -0.005)	0.025
	MET score	Beta	4.31 (-30.12 to 38.73)	0.806
	Current smoking	Odds ratio	0.95 (0.91 to 0.98)	0.005
	Current alcohol drinking	Odds ratio	0.98 (0.94 to 1.03)	0.390
LDL-C	Townsend deprivation index	Beta	-0.04 (-0.08 to 0.001)	0.057
	MET score	Beta	25.77 (-15.28 to 66.83)	0.219
	Current smoking	Odds ratio	0.93 (0.89 to 0.97)	0.002
	Current alcohol drinking	Odds ratio	0.97 (0.92 to 1.02)	0.247
TG	Townsend deprivation index	Beta	0.05 (0.01 to 0.08)	0.013
	MET score	Beta	28.04 (-9.54 to 65.62)	0.144
	Current smoking	Odds ratio	1.01 (0.97 to 1.05)	0.617
	Current alcohol drinking	Odds ratio	0.92 (0.87 to 0.96)	0.001

ApoB, apolipoprotein B; LDL-C, low-density lipoprotein cholesterol; MET, metabolic equivalent task; TG, triglycerides. Estimates are expressed as beta/odds ratio (95% confidence interval) per standard deviation increase in each lipid trait (approximately 0.24 g/L for apoB, 0.86 mmol/L for LDL-C, and 0.99 mmol/L for TG).

© 2024 Yang G et al. *JAMA Network Open.*

eTable 3. Univariable Mendelian Randomization Estimates for Genetically-Predicted ApoB, LDL-C, and TG on CAD, All-Cause Mortality, and Cause-Specific Mortality

Exposure	Outcome	Method	Odds ratio/ Hazard ratio	*P* value	*P* value (Egger intercept)
ApoB	CAD	IVW	1.66 (1.46 to 1.87)	<0.001	
	CAD	Weighted median	1.57 (1.42 to 1.74)	<0.001	
	CAD	MR Egger	1.74 (1.46 to 2.06)	<0.001	0.423
	CAD	Conmix	1.87 (1.68 to 2.01)	<0.001	
	All-cause mortality	IVW	1.11 (1.05 to 1.17)	<0.001	
	All-cause mortality	Weighted median	1.08 (1.00 to 1.17)	0.050	
	All-cause mortality	MR Egger	1.12 (1.04 to 1.20)	0.004	0.869
	All-cause mortality	Conmix	1.09 (1.04 to 1.14)	0.010	
	CVD mortality	IVW	1.37 (1.20 to 1.55)	<0.001	
	CVD mortality	Weighted median	1.43 (1.22 to 1.69)	<0.001	
	CVD mortality	MR Egger	1.54 (1.28 to 1.84)	<0.001	0.066
	CVD mortality	Conmix	1.40 (1.25 to 1.59)	<0.001	
	Cancer mortality	IVW	1.03 (0.96 to 1.10)	0.458	
	Cancer mortality	Weighted median	0.99 (0.90 to 1.09)	0.848	
	Cancer mortality	MR Egger	0.96 (0.87 to 1.05)	0.364	0.035
	Cancer mortality	Conmix	1.02 (0.92 to 1.08)	0.746	
	Non-CVD/cancer mortality	IVW	1.10 (0.99 to 1.21)	0.069	
	Non-CVD/cancer mortality	Weighted median	1.00 (0.87 to 1.15)	0.991	
	Non-CVD/cancer mortality	MR Egger	1.19 (1.03 to 1.36)	0.016	0.111
	Non-CVD/cancer mortality	Conmix	1.01 (0.91 to 1.12)	0.772	
LDL-C	CAD	IVW	1.73 (1.55 to 1.93)	<0.001	
	CAD	Weighted median	1.53 (1.38 to 1.71)	<0.001	
	CAD	MR Egger	1.83 (1.57 to 2.12)	<0.001	0.271
	CAD	Conmix	1.71 (1.61 to 1.83)	<0.001	
	All-cause mortality	IVW	1.15 (1.08 to 1.21)	<0.001	
	All-cause mortality	Weighted median	1.11 (1.01 to 1.22)	0.031	
	All-cause mortality	MR Egger	1.15 (1.06 to 1.24)	0.001	0.973
	All-cause mortality	Conmix	1.13 (1.05 to 1.20)	<0.001	
	CVD mortality	IVW	1.47 (1.29 to 1.67)	<0.001	
	CVD mortality	Weighted median	1.61 (1.31 to 1.97)	<0.001	
	CVD mortality	MR Egger	1.56 (1.31 to 1.86)	<0.001	0.332
	CVD mortality	Conmix	1.45 (1.28 to 1.63)	<0.001	
	Cancer mortality	IVW	1.04 (0.96 to 1.12)	0.301	
	Cancer mortality	Weighted median	0.99 (0.89 to 1.12)	0.926	
	Cancer mortality	MR Egger	1.00 (0.90 to 1.10)	0.941	0.203
	Cancer mortality	Conmix	1.02 (0.95 to 1.10)	0.527	
	Non-CVD/cancer mortality	IVW	1.15 (1.03 to 1.29)	0.015	
	Non-CVD/cancer mortality	Weighted median	1.08 (0.92 to 1.28)	0.357	
	Non-CVD/cancer mortality	MR Egger	1.19 (1.03 to 1.38)	0.022	0.471

© 2024 Yang G et al. *JAMA Network Open.*

	Non-CVD/cancer mortality	Conmix	1.08 (0.97 to 1.20)	0.114	
TG	CAD	IVW	1.59 (1.47 to 1.73)	<0.001	
	CAD	Weighted median	1.49 (1.35 to 1.64)	<0.001	
	CAD	MR Egger	1.37 (1.23 to 1.53)	<0.001	<0.001
	CAD	Conmix	1.65 (1.51 to 1.81)	<0.001	
	All-cause mortality	IVW	1.07 (1.01 to 1.13)	0.015	
	All-cause mortality	Weighted median	1.00 (0.92 to 1.09)	0.973	
	All-cause mortality	MR Egger	1.03 (0.95 to 1.11)	0.512	0.144
	All-cause mortality	Conmix	1.06 (1.00 to 1.10)	0.031	
	CVD mortality	IVW	1.19 (1.06 to 1.34)	0.003	
	CVD mortality	Weighted median	1.14 (0.95 to 1.37)	0.156	
	CVD mortality	MR Egger	1.08 (0.91 to 1.27)	0.376	0.104
	CVD mortality	Conmix	1.20 (1.07 to 1.34)	<0.001	
	Cancer mortality	IVW	1.02 (0.95 to 1.10)	0.559	
	Cancer mortality	Weighted median	0.99 (0.88 to 1.10)	0.820	
	Cancer mortality	MR Egger	0.99 (0.89 to 1.09)	0.775	0.332
	Cancer mortality	Conmix	1.02 (0.96 to 1.08)	0.716	
	Non-CVD/cancer mortality	IVW	1.09 (0.99 to 1.22)	0.089	
	Non-CVD/cancer mortality	Weighted median	0.93 (0.80 to 1.10)	0.405	
	Non-CVD/cancer mortality	MR Egger	1.08 (0.93 to 1.25)	0.318	0.790
	Non-CVD/cancer mortality	Conmix	1.02 (0.93 to 1.12)	0.608	

ApoB, apolipoprotein B; CAD, coronary artery disease; Conmix, contamination mixture method; CVD, cardiovascular disease; IVW, inverse-variance weighted; LDL-C, low-density lipoprotein cholesterol; TG, triglycerides. Estimates are expressed as odds ratio (95% confidence interval) for CAD, or hazard ratio (95% confidence interval) for mortality outcomes per standard deviation increase in each lipid trait (approximately 0.24 g/L for apoB, 0.86 mmol/L for LDL-C, and 0.99 mmol/L for TG).

© 2024 Yang G et al. *JAMA Network Open.*

eTable 4. Multivariable Mendelian Randomization Estimates for Genetically-Predicted ApoB, LDL-C, and TG on CAD, All-Cause Mortality, and Cause-Specific Mortality Controlling for Other Lipid Traits and Possible Confounders

Exposure	Adjusted for	Outcome	Method	Odds ratio/Hazard ratio	*P* value	*P* value (Egger intercept)
ApoB	TG	CAD	IVW	1.58 (1.43 to 1.75)	<0.001	
ApoB	TG	CAD	MR Egger	1.55 (1.39 to 1.74)	<0.001	0.478
ApoB	TG and smoking	CAD	IVW	1.60 (1.45 to 1.76)	<0.001	
ApoB	TG and smoking	CAD	MR Egger	1.57 (1.41 to 1.76)	<0.001	0.565
ApoB	TG	All-cause mortality	IVW	1.13 (1.06 to 1.20)	<0.001	
ApoB	TG	All-cause mortality	MR Egger	1.11 (1.04 to 1.19)	0.002	0.199
ApoB	TG and smoking	All-cause mortality	IVW	1.14 (1.08 to 1.21)	<0.001	
ApoB	TG and smoking	All-cause mortality	MR Egger	1.13 (1.05 to 1.20)	<0.001	0.288
ApoB	TG	CVD mortality	IVW	1.40 (1.23 to 1.59)	<0.001	
ApoB	TG	CVD mortality	MR Egger	1.39 (1.21 to 1.61)	<0.001	0.872
ApoB	TG and smoking	CVD mortality	IVW	1.40 (1.23 to 1.59)	<0.001	
ApoB	TG and smoking	CVD mortality	MR Egger	1.39 (1.21 to 1.61)	<0.001	0.882
ApoB	TG	Cancer mortality	IVW	1.05 (0.97 to 1.13)	0.260	
ApoB	TG	Cancer mortality	MR Egger	1.02 (0.93 to 1.11)	0.735	0.118
ApoB	TG and smoking	Cancer mortality	IVW	1.06 (0.98 to 1.14)	0.159	
ApoB	TG and smoking	Cancer mortality	MR Egger	1.03 (0.94 to 1.12)	0.510	0.163
ApoB	TG	Non-CVD/cancer mortality	IVW	1.12 (1.00 to 1.25)	0.046	
ApoB	TG	Non-CVD/cancer mortality	MR Egger	1.11 (0.98 to 1.26)	0.090	0.812
ApoB	TG and smoking	Non-CVD/cancer mortality	IVW	1.14 (1.02 to 1.27)	0.020	
ApoB	TG and smoking	Non-CVD/cancer mortality	MR Egger	1.14 (1.01 to 1.29)	0.037	0.979
LDL-C	TG	CAD	IVW	1.64 (1.46 to 1.84)	<0.001	
LDL-C	TG	CAD	MR Egger	1.75 (1.53 to 2.00)	<0.001	0.082
LDL-C	TG and smoking	CAD	IVW	1.65 (1.47 to 1.85)	<0.001	
LDL-C	TG and smoking	CAD	MR Egger	1.77 (1.54 to 2.03)	<0.001	0.068
LDL-C	TG	All-cause mortality	IVW	1.15 (1.08 to 1.22)	<0.001	

© 2024 Yang G et al. *JAMA Network Open.*

LDL-C	TG	All-cause mortality	MR Egger	1.14 (1.06 to 1.23)	0.001	0.759
LDL-C	TG and smoking	All-cause mortality	IVW	1.16 (1.09 to 1.23)	<0.001	
LDL-C	TG and smoking	All-cause mortality	MR Egger	1.16 (1.07 to 1.25)	<0.001	0.887
LDL-C	TG	CVD mortality	IVW	1.44 (1.26 to 1.66)	<0.001	
LDL-C	TG	CVD mortality	MR Egger	1.51 (1.28 to 1.78)	<0.001	0.301
LDL-C	TG and smoking	CVD mortality	IVW	1.45 (1.26 to 1.66)	<0.001	
LDL-C	TG and smoking	CVD mortality	MR Egger	1.52 (1.29 to 1.79)	<0.001	0.295
LDL-C	TG	Cancer mortality	IVW	1.04 (0.96 to 1.13)	0.316	
LDL-C	TG	Cancer mortality	MR Egger	1.01 (0.91 to 1.11)	0.888	0.193
LDL-C	TG and smoking	Cancer mortality	IVW	1.05 (0.97 to 1.14)	0.224	
LDL-C	TG and smoking	Cancer mortality	MR Egger	1.02 (0.92 to 1.12)	0.706	0.234
LDL-C	TG	Non-CVD/cancer mortality	IVW	1.17 (1.04 to 1.33)	0.010	
LDL-C	TG	Non-CVD/cancer mortality	MR Egger	1.18 (1.02 to 1.37)	0.025	0.870
LDL-C	TG and smoking	Non-CVD/cancer mortality	IVW	1.19 (1.05 to 1.35)	0.005	
LDL-C	TG and smoking	Non-CVD/cancer mortality	MR Egger	1.21 (1.04 to 1.39)	0.012	0.765
TG	ApoB	CAD	IVW	1.28 (1.16 to 1.41)	<0.001	
TG	ApoB	CAD	MR Egger	1.12 (0.98 to 1.27)	0.088	0.002
TG	ApoB and alcohol drinking	CAD	IVW	1.26 (1.15 to 1.39)	<0.001	
TG	ApoB and alcohol drinking	CAD	MR Egger	1.13 (1.00 to 1.29)	0.059	0.011
TG	ApoB	All-cause mortality	IVW	0.99 (0.94 to 1.06)	0.854	
TG	ApoB	All-cause mortality	MR Egger	0.96 (0.88 to 1.04)	0.275	0.148
TG	ApoB and alcohol drinking	All-cause mortality	IVW	0.99 (0.93 to 1.05)	0.726	
TG	ApoB and alcohol drinking	All-cause mortality	MR Egger	0.96 (0.89 to 1.04)	0.330	0.280
TG	ApoB	CVD mortality	IVW	0.97 (0.86 to 1.11)	0.690	
TG	ApoB	CVD mortality	MR Egger	0.90 (0.76 to 1.07)	0.243	0.188
TG	ApoB and alcohol drinking	CVD mortality	IVW	0.97 (0.85 to 1.10)	0.621	
TG	ApoB and alcohol drinking	CVD mortality	MR Egger	0.91 (0.77 to 1.08)	0.272	0.270
TG	ApoB	Cancer mortality	IVW	1.01 (0.93 to 1.09)	0.813	
TG	ApoB	Cancer mortality	MR Egger	0.97 (0.87 to 1.07)	0.538	0.228
TG	ApoB and alcohol drinking	Cancer mortality	IVW	1.01 (0.93 to 1.09)	0.836	

© 2024 Yang G et al. *JAMA Network Open.*

TG	ApoB and alcohol drinking	Cancer mortality	MR Egger	0.97 (0.87 to 1.07)	0.548	0.249
TG	ApoB	Non-CVD/cancer mortality	IVW	0.99 (0.88 to 1.10)	0.791	
TG	ApoB	Non-CVD/cancer mortality	MR Egger	0.98 (0.84 to 1.13)	0.760	0.871
TG	ApoB and alcohol drinking	Non-CVD/cancer mortality	IVW	0.97 (0.87 to 1.09)	0.631	
TG	ApoB and alcohol drinking	Non-CVD/cancer mortality	MR Egger	0.99 (0.86 to 1.15)	0.895	0.727

ApoB, apolipoprotein B; CAD, coronary artery disease; CVD, cardiovascular disease; IVW, inverse-variance weighted; LDL-C, low-density lipoprotein cholesterol; TG, triglycerides. Estimates are expressed as odds ratio (95% confidence interval) for CAD, or hazard ratio (95% confidence interval) for mortality outcomes per standard deviation increase in each lipid trait (approximately 0.24 g/L for apoB, 0.86 mmol/L for LDL-C, and 0.99 mmol/L for TG).

© 2024 Yang G et al. *JAMA Network Open.*

eTable 5. Univariable Mendelian Randomization Estimates for Genetically-Predicted ApoB, LDL-C, and TG on Parental All-Cause Mortality

Exposure	Method	Hazard ratio	*P* value	*P* value (Egger intercept)
ApoB	IVW	1.16 (1.12 to 1.21)	<0.001	
	Weighted median	1.15 (1.10 to 1.19)	<0.001	
	MR Egger	1.21 (1.14 to 1.27)	<0.001	0.081
	Conmix	1.17 (1.14 to 1.19)	<0.001	
LDL-C	IVW	1.18 (1.13 to 1.23)	<0.001	
	Weighted median	1.15 (1.10 to 1.20)	<0.001	
	MR Egger	1.19 (1.13 to 1.26)	<0.001	0.494
	Conmix	1.16 (1.12 to 1.19)	<0.001	
TG	IVW	1.14 (1.10 to 1.17)	<0.001	
	Weighted median	1.11 (1.07 to 1.15)	<0.001	
	MR Egger	1.08 (1.03 to 1.13)	0.001	0.003
	Conmix	1.14 (1.12 to 1.16)	<0.001	

ApoB, apolipoprotein B; Conmix, contamination mixture method; IVW, inverse-variance weighted; LDL-C, low-density lipoprotein cholesterol; TG, triglycerides. Estimates are expressed as hazard ratio (95% confidence interval) per standard deviation increase in each lipid trait (approximately 0.24 g/L for apoB, 0.86 mmol/L for LDL-C, and 0.99 mmol/L for TG).

© 2024 Yang G et al. *JAMA Network Open.*

eTable 6. Multivariable Mendelian Randomization Estimates for Genetically-Predicted ApoB, LDL-C, and TG on Parental All-Cause Mortality Controlling for Other Lipid Traits and Possible Confounders

Exposure	Adjusted for	Method	Hazard ratio	*P* value	*P* value (Egger intercept)
ApoB	TG	IVW	1.17 (1.12 to 1.21)	<0.001	
ApoB	TG	MR Egger	1.17 (1.12 to 1.23)	<0.001	0.574
ApoB	TG and smoking	IVW	1.17 (1.13 to 1.22)	<0.001	
ApoB	TG and smoking	MR Egger	1.18 (1.13 to 1.23)	<0.001	0.434
LDL-C	TG	IVW	1.16 (1.11 to 1.21)	<0.001	
LDL-C	TG	MR Egger	1.19 (1.13 to 1.25)	<0.001	0.056
LDL-C	TG and smoking	IVW	1.16 (1.11 to 1.21)	<0.001	
LDL-C	TG and smoking	MR Egger	1.19 (1.14 to 1.25)	<0.001	0.042
TG	ApoB	IVW	1.04 (1.00 to 1.08)	0.038	
TG	ApoB	MR Egger	0.99 (0.95 to 1.04)	0.814	0.005
TG	ApoB and alcohol drinking	IVW	1.03 (1.00 to 1.07)	0.062	
TG	ApoB and alcohol drinking	MR Egger	1.00 (0.95 to 1.05)	0.933	0.020

ApoB, apolipoprotein B; IVW, inverse-variance weighted; LDL-C, low-density lipoprotein cholesterol; TG, triglycerides. Estimates are expressed as hazard ratio (95% confidence interval) per standard deviation increase in each lipid trait (approximately 0.24 g/L for apoB, 0.86 mmol/L for LDL-C, and 0.99 mmol/L for TG).

© 2024 Yang G et al. *JAMA Network Open.*

eFigure 1. Shapes of the Associations Of Genetically-Predicted ApoB, LDL-C, and TG With CAD, All-Cause Mortality, and Cause-Specific Mortality Overall

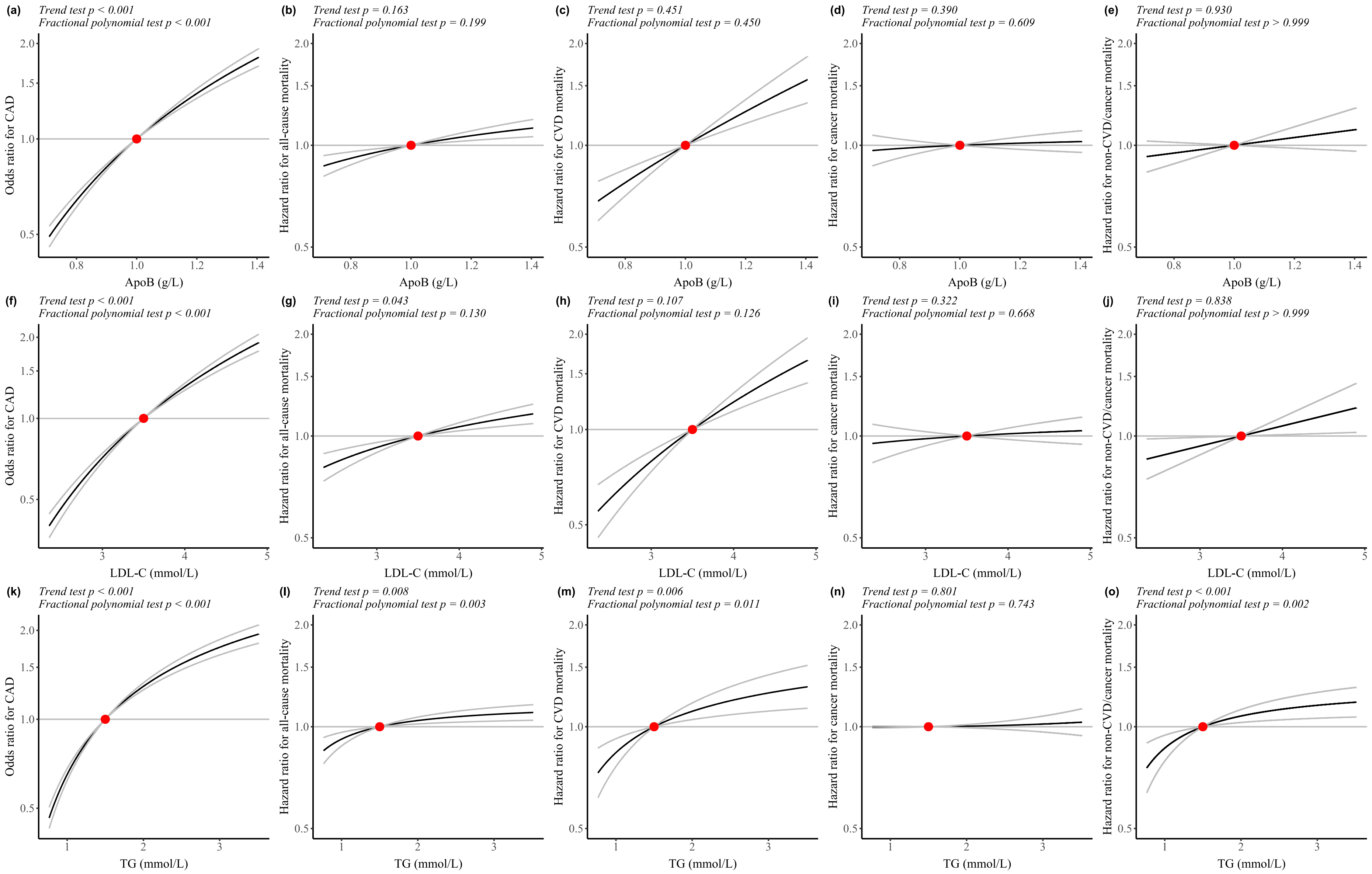

© 2024 Yang G et al. *JAMA Network Open.*

ApoB, apolipoprotein B; CAD, coronary artery disease; CVD, cardiovascular disease; LDL-C, low-density lipoprotein cholesterol; TG, triglycerides. The x-axis depicts apoB level in g/L, LDL-C level in mmol/L, or TG level in mmol/L. The y-axis depicts the odds ratio for CAD or hazard ratio for mortality outcomes with respect to the reference, plotted on a log scale. Reference is set to the median of each lipid trait (1.0 g/L for apoB, 3.5 mmol/L for LDL-C, and 1.5 mmol/L for TG). The black lines represent the dose-response relationship, and the grey lines represent the 95% confidence intervals. The trend test assesses whether a linear trend in the stratum-specific estimates exists, and the fractional polynomial test examines whether a non-linear model fits the exposure-outcome relationship better than a linear model.

© 2024 Yang G et al. *JAMA Network Open.*

eTable 7. Stratum-Specific Mendelian Randomization Estimates for Genetically-Predicted ApoB on CAD, All-Cause Mortality, and Cause-Specific Mortality

Stratum	Mean apoB (g/L)	Outcome	Odds ratio/Hazard ratio	*P* value
1	0.71	CAD	1.96 (1.75 to 2.21)	<0.001
2	0.81	CAD	2.29 (2.00 to 2.61)	<0.001
3	0.88	CAD	1.96 (1.69 to 2.27)	<0.001
4	0.94	CAD	1.69 (1.44 to 1.99)	<0.001
5	1.00	CAD	1.44 (1.21 to 1.72)	<0.001
6	1.05	CAD	1.36 (1.14 to 1.63)	0.001
7	1.11	CAD	1.32 (1.10 to 1.60)	0.003
8	1.18	CAD	1.44 (1.19 to 1.74)	<0.001
9	1.26	CAD	1.46 (1.20 to 1.77)	<0.001
10	1.41	CAD	1.20 (1.00 to 1.43)	0.047
1	0.71	All-cause mortality	1.17 (1.03 to 1.33)	0.013
2	0.81	All-cause mortality	1.10 (0.96 to 1.27)	0.150
3	0.88	All-cause mortality	1.16 (1.01 to 1.34)	0.038
4	0.94	All-cause mortality	1.11 (0.96 to 1.29)	0.152
5	1.00	All-cause mortality	0.97 (0.84 to 1.13)	0.712
6	1.05	All-cause mortality	1.06 (0.91 to 1.23)	0.450
7	1.11	All-cause mortality	1.12 (0.96 to 1.31)	0.151
8	1.18	All-cause mortality	1.29 (1.11 to 1.51)	0.001
9	1.26	All-cause mortality	1.01 (0.87 to 1.17)	0.935
10	1.41	All-cause mortality	1.01 (0.87 to 1.17)	0.897
1	0.71	CVD mortality	1.28 (1.00 to 1.65)	0.054
2	0.81	CVD mortality	1.65 (1.23 to 2.20)	0.001
3	0.88	CVD mortality	1.45 (1.07 to 1.97)	0.018
4	0.94	CVD mortality	1.71 (1.21 to 2.40)	0.002

© 2024 Yang G et al. *JAMA Network Open.*

5	1.00	CVD mortality	1.08 (0.77 to 1.51)	0.659
6	1.05	CVD mortality	1.36 (0.96 to 1.90)	0.080
7	1.11	CVD mortality	1.09 (0.76 to 1.56)	0.648
8	1.18	CVD mortality	1.46 (1.01 to 2.10)	0.043
9	1.26	CVD mortality	1.17 (0.83 to 1.64)	0.362
10	1.41	CVD mortality	1.35 (0.96 to 1.89)	0.084
1	0.71	Cancer mortality	1.08 (0.89 to 1.30)	0.448
2	0.81	Cancer mortality	0.97 (0.80 to 1.18)	0.747
3	0.88	Cancer mortality	1.16 (0.95 to 1.42)	0.158
4	0.94	Cancer mortality	0.96 (0.78 to 1.18)	0.692
5	1.00	Cancer mortality	0.98 (0.80 to 1.21)	0.873
6	1.05	Cancer mortality	0.98 (0.80 to 1.20)	0.850
7	1.11	Cancer mortality	1.07 (0.87 to 1.32)	0.519
8	1.18	Cancer mortality	1.21 (0.98 to 1.49)	0.071
9	1.26	Cancer mortality	0.91 (0.75 to 1.11)	0.344
10	1.41	Cancer mortality	0.96 (0.79 to 1.17)	0.677
1	0.71	Non-CVD/cancer mortality	1.18 (0.94 to 1.48)	0.145
2	0.81	Non-CVD/cancer mortality	0.99 (0.77 to 1.27)	0.908
3	0.88	Non-CVD/cancer mortality	0.97 (0.74 to 1.27)	0.822
4	0.94	Non-CVD/cancer mortality	1.07 (0.81 to 1.42)	0.650
5	1.00	Non-CVD/cancer mortality	0.85 (0.64 to 1.12)	0.244
6	1.05	Non-CVD/cancer mortality	1.00 (0.74 to 1.34)	0.998
7	1.11	Non-CVD/cancer mortality	1.26 (0.93 to 1.72)	0.135
8	1.18	Non-CVD/cancer mortality	1.39 (1.03 to 1.88)	0.033
9	1.26	Non-CVD/cancer mortality	1.15 (0.83 to 1.57)	0.402
10	1.41	Non-CVD/cancer mortality	0.91 (0.67 to 1.23)	0.533

ApoB, apolipoprotein B; CAD, coronary artery disease; CVD, cardiovascular disease. Estimates are expressed as odds ratio (95% confidence interval) for CAD, or hazard ratio (95% confidence interval) for mortality outcomes per standard deviation increase in apoB (approximately 0.24 g/L).

© 2024 Yang G et al. *JAMA Network Open.*

eTable 8. Stratum-Specific Mendelian Randomization Estimates for Genetically-Predicted LDL-C on CAD, All-Cause Mortality, and Cause-Specific Mortality

Stratum	Mean LDL-C (mmol/L)	Outcome	Odds ratio/Hazard ratio	*P* value
1	2.35	CAD	2.38 (2.11 to 2.69)	<0.001
2	2.74	CAD	2.36 (2.04 to 2.73)	<0.001
3	3.01	CAD	2.18 (1.84 to 2.59)	<0.001
4	3.24	CAD	2.27 (1.87 to 2.75)	<0.001
5	3.45	CAD	1.37 (1.12 to 1.69)	0.003
6	3.65	CAD	1.77 (1.42 to 2.21)	<0.001
7	3.86	CAD	1.31 (1.04 to 1.66)	0.024
8	4.10	CAD	1.54 (1.22 to 1.94)	<0.001
9	4.41	CAD	1.23 (0.97 to 1.55)	0.086
10	4.90	CAD	1.34 (1.08 to 1.67)	0.008
1	2.35	All-cause mortality	1.08 (0.95 to 1.24)	0.241
2	2.74	All-cause mortality	1.23 (1.05 to 1.43)	0.008
3	3.01	All-cause mortality	1.24 (1.05 to 1.47)	0.010
4	3.24	All-cause mortality	1.29 (1.08 to 1.53)	0.004
5	3.45	All-cause mortality	1.06 (0.89 to 1.27)	0.500
6	3.65	All-cause mortality	1.30 (1.09 to 1.56)	0.004
7	3.86	All-cause mortality	1.09 (0.91 to 1.31)	0.339
8	4.10	All-cause mortality	1.04 (0.87 to 1.25)	0.644
9	4.41	All-cause mortality	1.14 (0.95 to 1.38)	0.160
10	4.90	All-cause mortality	0.99 (0.83 to 1.17)	0.897
1	2.35	CVD mortality	1.63 (1.24 to 2.12)	<0.001
2	2.74	CVD mortality	1.86 (1.36 to 2.56)	<0.001
3	3.01	CVD mortality	1.45 (1.01 to 2.09)	0.044
4	3.24	CVD mortality	1.96 (1.32 to 2.89)	0.001
5	3.45	CVD mortality	0.88 (0.59 to 1.31)	0.526
6	3.65	CVD mortality	2.09 (1.38 to 3.15)	<0.001

© 2024 Yang G et al. *JAMA Network Open.*

7	3.86	CVD mortality	1.21 (0.80 to 1.82)	0.371
8	4.10	CVD mortality	1.36 (0.88 to 2.09)	0.165
9	4.41	CVD mortality	1.59 (1.01 to 2.50)	0.044
10	4.90	CVD mortality	1.15 (0.77 to 1.71)	0.489
1	2.35	Cancer mortality	0.85 (0.69 to 1.04)	0.108
2	2.74	Cancer mortality	1.10 (0.88 to 1.36)	0.411
3	3.01	Cancer mortality	1.17 (0.93 to 1.49)	0.181
4	3.24	Cancer mortality	1.17 (0.92 to 1.50)	0.197
5	3.45	Cancer mortality	1.21 (0.95 to 1.54)	0.127
6	3.65	Cancer mortality	1.03 (0.81 to 1.31)	0.788
7	3.86	Cancer mortality	1.02 (0.80 to 1.31)	0.866
8	4.10	Cancer mortality	0.96 (0.75 to 1.22)	0.732
9	4.41	Cancer mortality	0.98 (0.77 to 1.25)	0.886
10	4.90	Cancer mortality	0.96 (0.77 to 1.21)	0.752
1	2.35	Non-CVD/cancer mortality	1.10 (0.87 to 1.40)	0.428
2	2.74	Non-CVD/cancer mortality	1.04 (0.78 to 1.38)	0.803
3	3.01	Non-CVD/cancer mortality	1.14 (0.83 to 1.56)	0.410
4	3.24	Non-CVD/cancer mortality	1.15 (0.83 to 1.59)	0.392
5	3.45	Non-CVD/cancer mortality	0.92 (0.66 to 1.29)	0.636
6	3.65	Non-CVD/cancer mortality	1.56 (1.09 to 2.23)	0.016
7	3.86	Non-CVD/cancer mortality	1.19 (0.83 to 1.72)	0.342
8	4.10	Non-CVD/cancer mortality	1.03 (0.72 to 1.47)	0.872
9	4.41	Non-CVD/cancer mortality	1.29 (0.89 to 1.88)	0.185
10	4.90	Non-CVD/cancer mortality	0.98 (0.69 to 1.40)	0.915

CAD, coronary artery disease; CVD, cardiovascular disease; LDL-C, low-density lipoprotein cholesterol. Estimates are expressed as odds ratio (95% confidence interval) for CAD, or hazard ratio (95% confidence interval) for mortality outcomes per standard deviation increase in LDL-C (approximately 0.86 mmol/L).

© 2024 Yang G et al. *JAMA Network Open.*

eTable 9. Stratum-Specific Mendelian Randomization Estimates for Genetically-Predicted TG on CAD, All-Cause Mortality, and Cause-Specific Mortality

Stratum	Mean TG (mmol/L)	Outcome	Odds ratio/Hazard ratio	*P* value
1	0.76	CAD	8.78 (6.17 to 12.50)	<0.001
2	0.95	CAD	3.43 (2.51 to 4.70)	<0.001
3	1.11	CAD	2.91 (2.16 to 3.92)	<0.001
4	1.27	CAD	2.82 (2.12 to 3.75)	<0.001
5	1.44	CAD	1.82 (1.38 to 2.40)	<0.001
6	1.64	CAD	1.57 (1.20 to 2.04)	0.001
7	1.87	CAD	1.59 (1.22 to 2.06)	<0.001
8	2.17	CAD	1.37 (1.07 to 1.76)	0.012
9	2.62	CAD	1.35 (1.05 to 1.73)	0.021
10	3.51	CAD	1.15 (0.91 to 1.45)	0.238
1	0.76	All-cause mortality	2.78 (2.05 to 3.75)	<0.001
2	0.95	All-cause mortality	0.86 (0.65 to 1.13)	0.279
3	1.11	All-cause mortality	1.25 (0.96 to 1.64)	0.100
4	1.27	All-cause mortality	1.19 (0.92 to 1.55)	0.183
5	1.44	All-cause mortality	1.10 (0.85 to 1.42)	0.471
6	1.64	All-cause mortality	1.15 (0.90 to 1.47)	0.276
7	1.87	All-cause mortality	1.08 (0.84 to 1.40)	0.529
8	2.17	All-cause mortality	1.04 (0.82 to 1.31)	0.772
9	2.62	All-cause mortality	1.05 (0.82 to 1.34)	0.682
10	3.51	All-cause mortality	0.99 (0.79 to 1.24)	0.917
1	0.76	CVD mortality	2.37 (1.18 to 4.75)	0.015
2	0.95	CVD mortality	0.75 (0.40 to 1.42)	0.379
3	1.11	CVD mortality	3.24 (1.75 to 6.02)	<0.001
4	1.27	CVD mortality	1.14 (0.64 to 2.02)	0.664
5	1.44	CVD mortality	1.40 (0.79 to 2.47)	0.253
6	1.64	CVD mortality	1.37 (0.79 to 2.37)	0.259

© 2024 Yang G et al. *JAMA Network Open.*

7	1.87	CVD mortality	1.36 (0.79 to 2.34)	0.262
8	2.17	CVD mortality	1.04 (0.62 to 1.72)	0.889
9	2.62	CVD mortality	1.17 (0.69 to 2.00)	0.561
10	3.51	CVD mortality	1.00 (0.62 to 1.60)	0.985
1	0.76	Cancer mortality	2.56 (1.66 to 3.95)	<0.001
2	0.95	Cancer mortality	0.56 (0.38 to 0.82)	0.003
3	1.11	Cancer mortality	1.02 (0.70 to 1.48)	0.912
4	1.27	Cancer mortality	1.05 (0.73 to 1.51)	0.800
5	1.44	Cancer mortality	1.02 (0.72 to 1.44)	0.925
6	1.64	Cancer mortality	1.10 (0.78 to 1.56)	0.585
7	1.87	Cancer mortality	1.00 (0.70 to 1.42)	0.985
8	2.17	Cancer mortality	1.02 (0.73 to 1.42)	0.921
9	2.62	Cancer mortality	0.97 (0.69 to 1.35)	0.859
10	3.51	Cancer mortality	1.04 (0.75 to 1.44)	0.811
1	0.76	Non-CVD/cancer mortality	3.47 (2.03 to 5.93)	<0.001
2	0.95	Non-CVD/cancer mortality	2.17 (1.28 to 3.66)	0.004
3	1.11	Non-CVD/cancer mortality	1.05 (0.62 to 1.76)	0.860
4	1.27	Non-CVD/cancer mortality	1.58 (0.96 to 2.57)	0.070
5	1.44	Non-CVD/cancer mortality	1.13 (0.69 to 1.87)	0.627
6	1.64	Non-CVD/cancer mortality	1.08 (0.67 to 1.74)	0.739
7	1.87	Non-CVD/cancer mortality	1.12 (0.68 to 1.84)	0.662
8	2.17	Non-CVD/cancer mortality	1.06 (0.67 to 1.69)	0.797
9	2.62	Non-CVD/cancer mortality	1.15 (0.71 to 1.87)	0.574
10	3.51	Non-CVD/cancer mortality	0.88 (0.57 to 1.38)	0.585

CAD, coronary artery disease; CVD, cardiovascular disease; TG, triglycerides. Estimates are expressed as odds ratio (95% confidence interval) for CAD, or hazard ratio (95% confidence interval) for mortality outcomes per standard deviation increase in TG (approximately 0.99 mmol/L).

© 2024 Yang G et al. *JAMA Network Open.*

eFigure 2. Shapes of the Associations of Genetically-Predicted ApoB, LDL-C, and TG With CAD, All-Cause Mortality, and Cause-Specific Mortality for Males

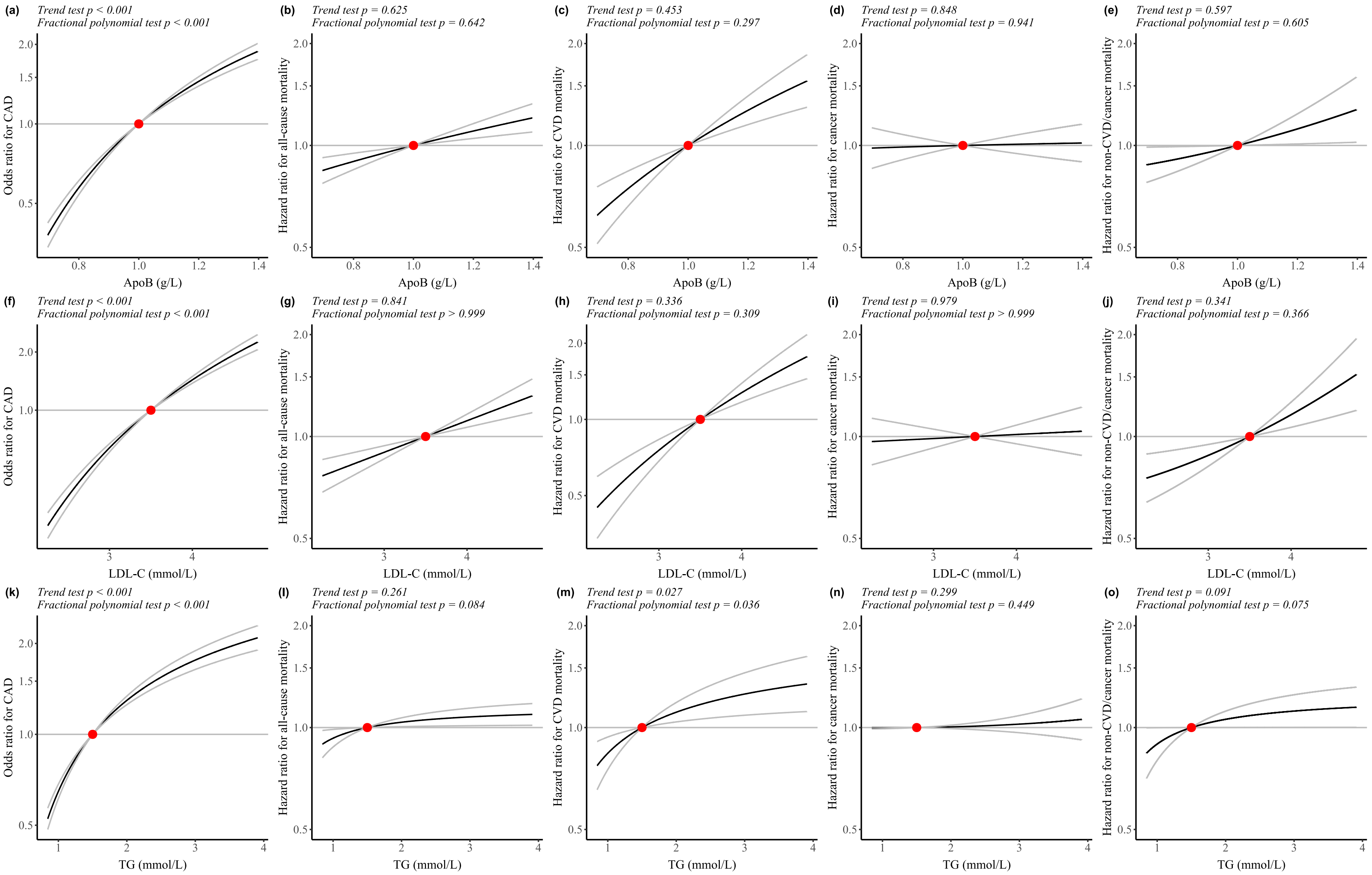

© 2024 Yang G et al. *JAMA Network Open.*

ApoB, apolipoprotein B; CAD, coronary artery disease; CVD, cardiovascular disease; LDL-C, low-density lipoprotein cholesterol; TG, triglycerides. The x-axis depicts apoB level in g/L, LDL-C level in mmol/L, or TG level in mmol/L. The y-axis depicts the odds ratio for CAD or hazard ratio for mortality outcomes with respect to the reference, plotted on a log scale. Reference is set to the median of each lipid trait (1.0 g/L for apoB, 3.5 mmol/L for LDL-C, and 1.5 mmol/L for TG). The black lines represent the dose-response relationship, and the grey lines represent the 95% confidence intervals. The trend test assesses whether a linear trend in the stratum-specific estimates exists, and the fractional polynomial test examines whether a non-linear model fits the exposure-outcome relationship better than a linear model.

© 2024 Yang G et al. *JAMA Network Open.*

eFigure 3. Shapes of the Associations of Genetically-Predicted ApoB, LDL-C, and TG With CAD, All-Cause Mortality, and Cause-Specific Mortality for Females

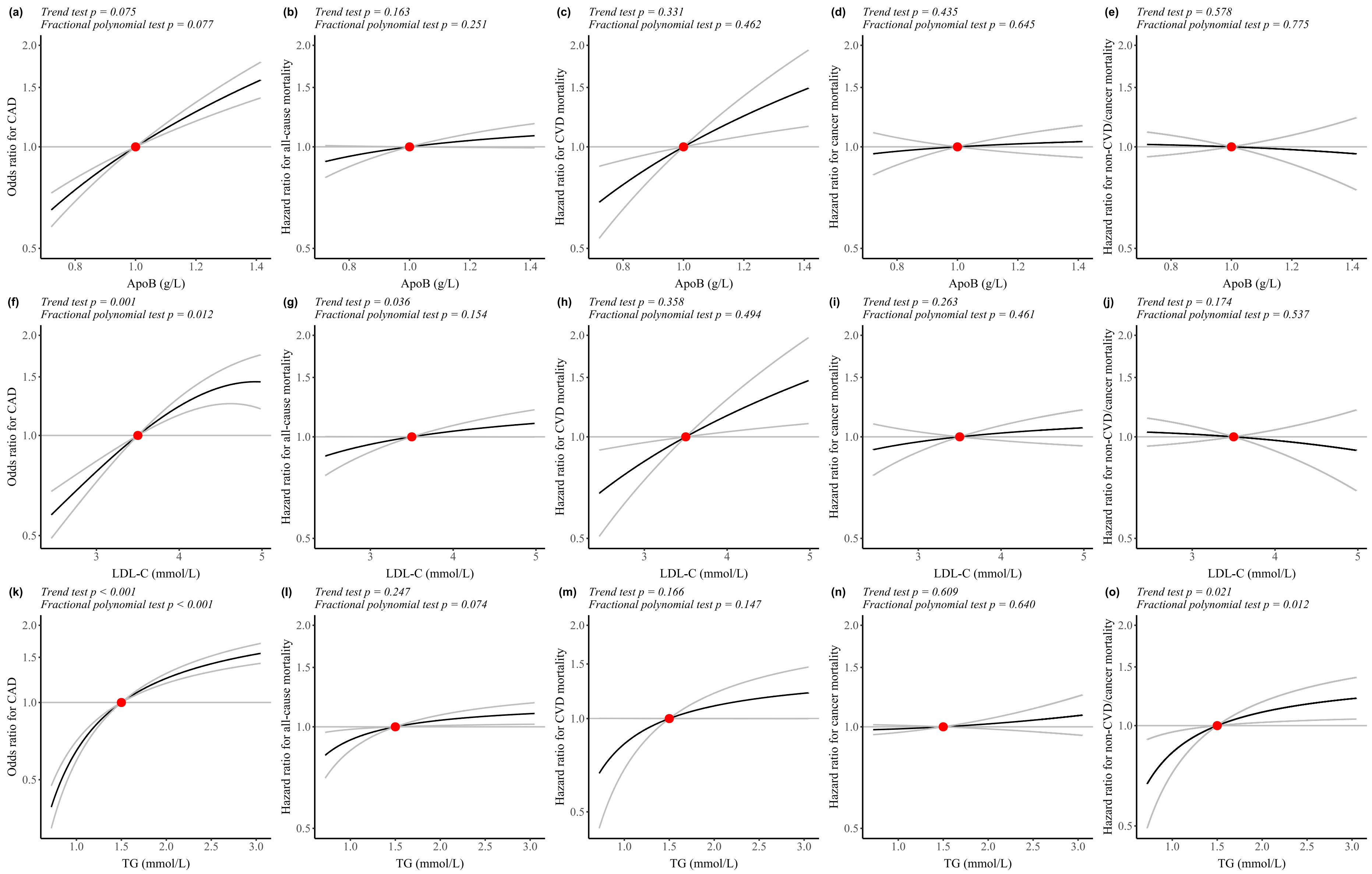

© 2024 Yang G et al. *JAMA Network Open.*

ApoB, apolipoprotein B; CAD, coronary artery disease; CVD, cardiovascular disease; LDL-C, low-density lipoprotein cholesterol; TG, triglycerides. The x-axis depicts apoB level in g/L, LDL-C level in mmol/L, or TG level in mmol/L. The y-axis depicts the odds ratio for CAD or hazard ratio for mortality outcomes with respect to the reference, plotted on a log scale. Reference is set to the median of each lipid trait (1.0 g/L for apoB, 3.5 mmol/L for LDL-C, and 1.5 mmol/L for TG). The black lines represent the dose-response relationship, and the grey lines represent the 95% confidence intervals. The trend test assesses whether a linear trend in the stratum-specific estimates exists, and the fractional polynomial test examines whether a non-linear model fits the exposure-outcome relationship better than a linear model.

© 2024 Yang G et al. *JAMA Network Open.*

eFigure 4. Shapes of the Associations of Genetically-Predicted ApoB, LDL-C, and TG With CAD, All-Cause Mortality, and Cause-Specific Mortality for Younger People

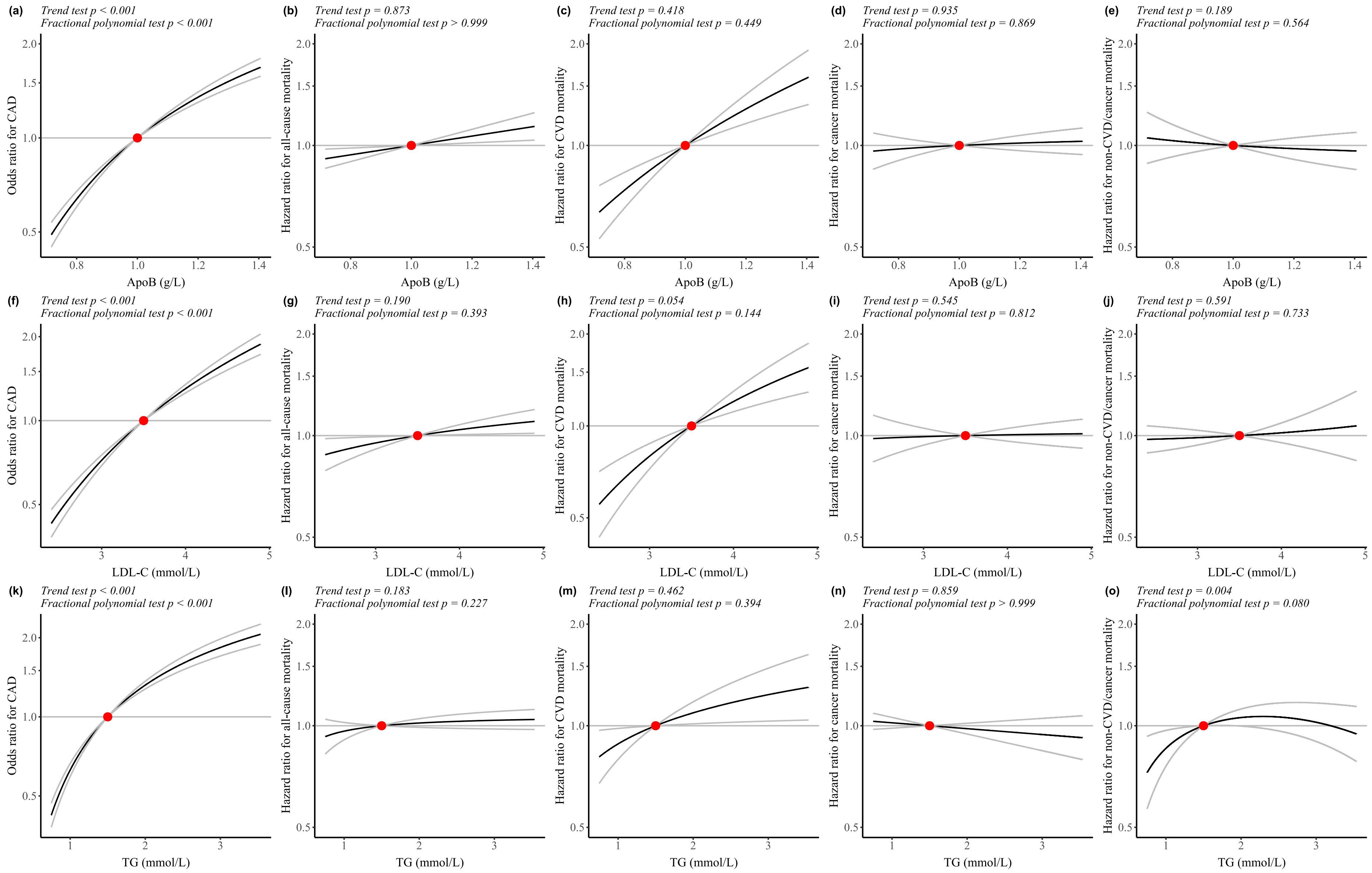

© 2024 Yang G et al. *JAMA Network Open.*

ApoB, apolipoprotein B; CAD, coronary artery disease; CVD, cardiovascular disease; LDL-C, low-density lipoprotein cholesterol; TG, triglycerides. The x-axis depicts apoB level in g/L, LDL-C level in mmol/L, or TG level in mmol/L. The y-axis depicts the odds ratio for CAD or hazard ratio for mortality outcomes with respect to the reference, plotted on a log scale. Reference is set to the median of each lipid trait (1.0 g/L for apoB, 3.5 mmol/L for LDL-C, and 1.5 mmol/L for TG). The black lines represent the dose-response relationship, and the grey lines represent the 95% confidence intervals. The trend test assesses whether a linear trend in the stratum-specific estimates exists, and the fractional polynomial test examines whether a non-linear model fits the exposure-outcome relationship better than a linear model.

© 2024 Yang G et al. *JAMA Network Open.*

eFigure 5. Shapes of the Associations of Genetically-Predicted ApoB, LDL-C, and TG With CAD, All-Cause Mortality, and Cause-Specific Mortality for Older People

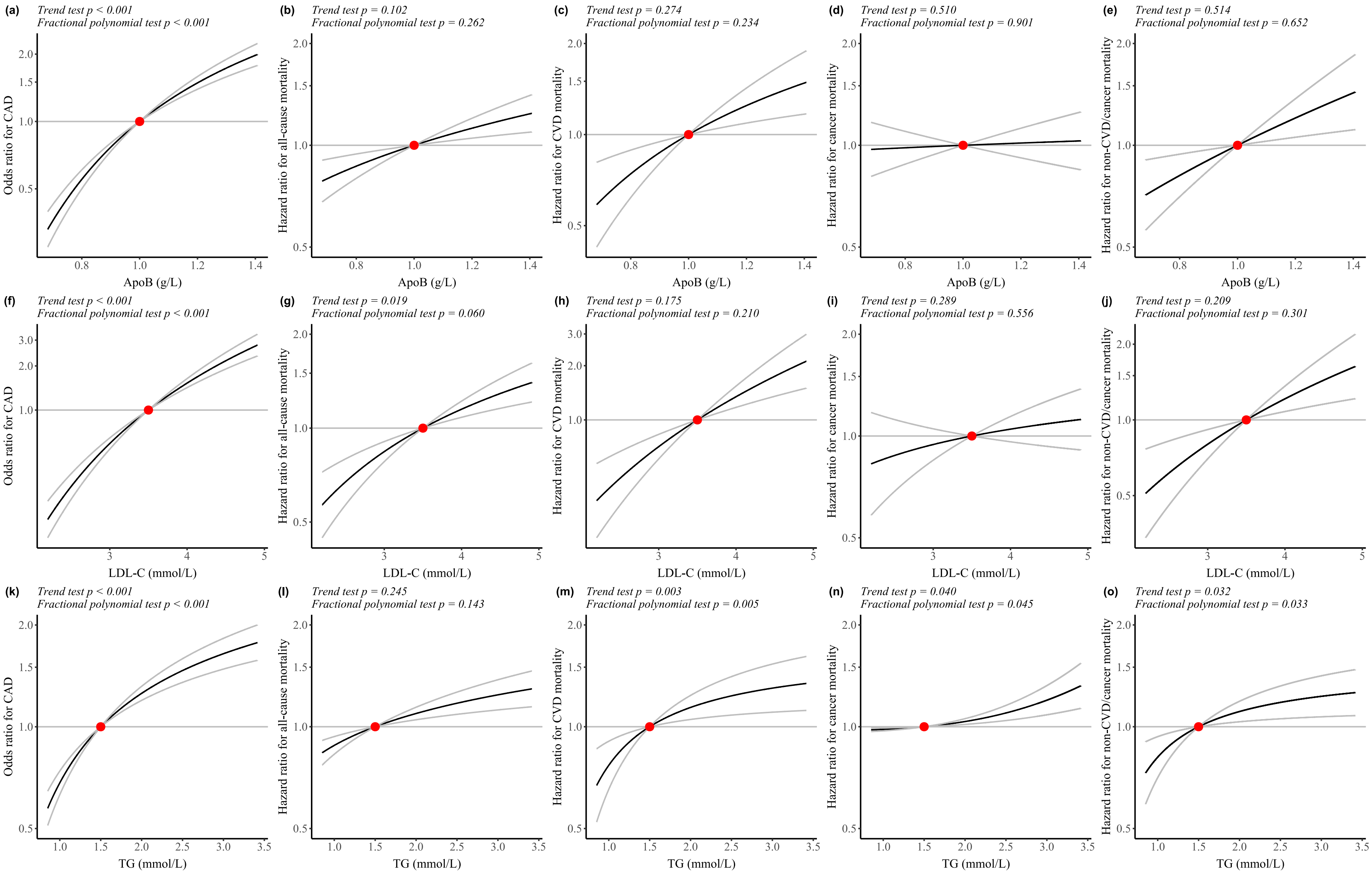

© 2024 Yang G et al. *JAMA Network Open.*

ApoB, apolipoprotein B; CAD, coronary artery disease; CVD, cardiovascular disease; LDL-C, low-density lipoprotein cholesterol; TG, triglycerides. The x-axis depicts apoB level in g/L, LDL-C level in mmol/L, or TG level in mmol/L. The y-axis depicts the odds ratio for CAD or hazard ratio for mortality outcomes with respect to the reference, plotted on a log scale. Reference is set to the median of each lipid trait (1.0 g/L for apoB, 3.5 mmol/L for LDL-C, and 1.5 mmol/L for TG). The black lines represent the dose-response relationship, and the grey lines represent the 95% confidence intervals. The trend test assesses whether a linear trend in the stratum-specific estimates exists, and the fractional polynomial test examines whether a non-linear model fits the exposure-outcome relationship better than a linear model.

© 2024 Yang G et al. *JAMA Network Open.*
