# Supplementary material for: Dose-Response Associations of Lipid Traits With Coronary Artery Disease and Mortality
Source: JAMA Netw Open. 2024 Jan 19;7(1):e2352572. doi: 10.1001/jamanetworkopen.2023.52572 (PMC10799266; doi:10.1001/jamanetworkopen.2023.52572)
Supplement: Supplement 2. — Data Sharing Statement [file jamanetwopen-e2352572-s002.pdf]

Data Sharing Statement

Yang. Dose-Response Associations of Lipid Traits With Coronary Artery Disease and Mortality. *JAMA Netw Open*. Published January 19, 2024. doi:10.1001/jamanetworkopen.2023.52572

Data
Data available: No

Additional Information
Explanation for why data not available: Data from UK Biobank are available to any bona fide scientific research on application. We do not own UK Biobank data, and so we cannot release these data into the public domain ourselves.
